# A Requirement for Zic2 in the Regulation of Nodal Expression Underlies the Establishment of Left-Sided Identity

**DOI:** 10.1038/s41598-018-28714-1

**Published:** 2018-07-11

**Authors:** Iain M. Dykes, Dorota Szumska, Linta Kuncheria, Rathi Puliyadi, Chiann-mun Chen, Costis Papanayotou, Helen Lockstone, Christèle Dubourg, Véronique David, Jurgen E. Schneider, Thomas M. Keane, David J. Adams, Steve D. M. Brown, Sandra Mercier, Sylvie Odent, Jérôme Collignon, Shoumo Bhattacharya

**Affiliations:** 1 Department of Cardiovascular Medicine, BHF Centre of Research Excellence, University of Oxford, Roosevelt Drive, Headington, Oxford, OX3 7BN United Kingdom; 2 Wellcome Trust Centre for Human Genetics, University of Oxford, Roosevelt Drive, Headington, Oxford, OX3 7BN United Kingdom; 3 Institut Jacques Monod, UMR 7592, CNRS, Université Paris-Diderot, Sorbonne Paris Cité, 75013 Paris, France; 4 Faculté de Médecine, Institut de Génétique et Développement de Rennes, UMR 6290, Université de Rennes 1, Rennes, France; 5 Laboratoire de Génétique Moléculaire, CHU Rennes, Rennes, France; 6 Wellcome Trust Sanger Institute, Wellcome Trust Genome Campus, Hinxton, Cambridge CB10 1SA United Kingdom; 7 Mammalian Genetics Unit, MRC Harwell Institute, Harwell Science and Innovation Campus, Oxfordshire, OX11 0RD United Kingdom; 8 CHU Nantes, Service de Génétique Médicale, 44093 Nantes, France; 9 Service de Génétique clinique, CHU Rennes, France; 10 Present Address: School of Pharmacy and Biomolecular Sciences, Liverpool John Moores University, Byrom Street, Liverpool, L3 3AF United Kingdom; 11 Present Address: Center of Basic Research, Biomedical Research Foundation Academy of Athens, Athens, 11527 Greece

## Abstract

*ZIC2* mutation is known to cause holoprosencephaly (HPE). A subset of *ZIC2* HPE probands harbour cardiovascular and visceral anomalies suggestive of laterality defects. 3D-imaging of novel mouse *Zic2* mutants uncovers, in addition to HPE, laterality defects in lungs, heart, vasculature and viscera. A strong bias towards right isomerism indicates a failure to establish left identity in the lateral plate mesoderm (LPM), a phenotype that cannot be explained simply by the defective ciliogenesis previously noted in Zic2 mutants. Gene expression analysis showed that the left-determining NODAL-dependent signalling cascade fails to be activated in the LPM, and that the expression of *Nodal* at the node, which normally triggers this event, is itself defective in these embryos. Analysis of ChiP-seq data, *in vitro* transcriptional assays and mutagenesis reveals a requirement for a low-affinity ZIC2 binding site for the activation of the *Nodal* enhancer HBE, which is normally active in node precursor cells. These data show that ZIC2 is required for correct *Nodal* expression at the node and suggest a model in which ZIC2 acts at different levels to establish LR asymmetry, promoting both the production of the signal that induces left side identity and the morphogenesis of the cilia that bias its distribution.

## Introduction

ZIC2 mutation is one of four principal genetic risk factors for holoprosencephaly (HPE), the most common structural brain disease^1–3^. A similar HPE phenotype is seen in mice homozygous either for the ethylnitrosourea (ENU) mutant allele *Zic2*^*ku*^ ^4^ or a hypomorphic allele, *Zic2*^*tm1Jaru*^ ^5^. The midline organiser known as the node, which forms at the leading edge of the primitive streak at the end of gastrulation, plays a central role in the generation of mesendodermal structures required to pattern the neural tube, such as the pre-chordal plate (PCP) and the notochord. *Zic2* is transiently expressed at the node from E7.0 to E7.5^6^. Development of the PCP is impaired in the *Zic2*^*ku*^ mutant at the late-streak stage^4^. *Nodal*, a member of the TGFβ family which signals via SMAD2, 3, is expressed in the epiblast giving rise to the primitive streak and in the primitive streak itself. It is required for primitive streak formation as well as for that of the node and of node-derived mesendoderm^7^. An epistatic genetic interaction exists between *Nodal* and *Zic2* in forebrain development^8^.

Laterality of the heart and viscera is also determined by the NODAL pathway. Bilateral symmetry is broken through generation of a leftward fluid flow by cilia within the node^9,10^. *Nodal* is initially expressed bilaterally at the node within perinodal cells (at E7.5 in mouse), but in response to the nodal flow, this expression is reinforced in left perinodal cells, thus becoming asymmetric^11,12^. NODAL produced in perinodal cells is required to trigger its own expression in the left lateral plate mesoderm (LPM)^13^, where it induces the expression of *Lefty2*^14^, a NODAL antagonist, and *Pitx2c*^15,16^. *Pitx2c* expression in the left LPM determines left-sided identity in mesoderm derivatives. In the absence of *Pitx2c* expression the default pattern is that of two morphologically right sides, a condition known as right-isomerism. Ectopic activation of *Pitx2c* in the right lateral mesoderm only results in *situs inversus* whereas bilateral expression of *Pitx2c* results in two morphologically left sides, known as left-isomerism. These phenotypes can be distinguished by examination of lung lobulation, atrial morphology and other characters^17^. The establishment of Left-Right (LR) polarity is essential for key aspects of cardiovascular, thoracic and abdominal development. It determines the lateralised identity of atria and lungs, influences looping morphogenesis of the linear heart tube and the gut, directs asymmetric remodelling of the vascular system and determines the positioning of visceral organs such as stomach and pancreas^17–21^. Mutations mapping to NODAL pathway genes are associated with heterotaxy^22,23^, a condition characterized by discordant LR arrangement of internal organs which accounts for approximately 3% of all congenital heart disease^22^.

Cardiac malformations have been noted amongst a number of extra-craniofacial anomalies reported in HPE patients with *ZIC2* mutation, occurring in 9–14% of probands^2,3^, but have not previously been described in detail. We hypothesise these may result from an underlying laterality defect. The *Zic2*^*ku*^ mutant shows randomised direction of heart tube looping during cardiac morphogenesis^24^, supporting this hypothesis. These embryos exhibit mid-gestation lethality, preventing a more detailed study of laterality defects^24^. Nodal cilia are shorter and morphologically abnormal in the *Zic2*^*ku*^ mutant suggesting that *Zic2* may function during cilia morphogenesis^24^. Expression of *Nodal* at the node and of downstream genes in the LPM is reduced in the *Zic2*^*ku*^ mutant^24^, but ectopic right-sided or bilateral expression, which is present in *iv* embryos with nodal cilia defects^12,25^, is not observed.

*Zic2* is also expressed earlier in development and is present in both embryonic (ESC) and epiblast (EpiSC) stem cells. ZIC2 is bound to the *Nodal* locus in both cell types^26,27^. EpiSCs have been shown to resemble cells from the anterior primitive streak^28^, from which the node is derived. ZIC2 appears to play a central role in the transition from the naive pluripotent state of ESCs to the primed pluripotent state of EpiSCs^26,29^ and has been proposed to act as a “pioneer factor” that functions to seed enhancers, recruiting additional transcription factors in order to prime loci for transcriptional activation at a later stage^26^. The MDB3-NURD chromatin remodelling complex is associated with ZIC2 at a subset of binding sites in ESCs^27^, this complex is known to play a role in fine-tuning dynamic expression of bivalent enhancers during development^30^, consistent with a role for ZIC2 in such a priming process.

Here, we examine clinical data and show that the cardiovascular, pulmonary and visceral phenotypes of *ZIC2* HPE patients are consistent with an underlying laterality defect. We identify a novel *Zic2* ENU mouse mutant in a screen for cardiovascular laterality phenotypes. We use 3D imaging to characterise in detail the phenotype of this mutant and that of a series of *Zic2* alleles generated by TALEN gene editing^31^, revealing a complex set of cardiovascular, thoracic and abdominal malformations, in addition to the previously-described holoprosencephaly and heart tube looping phenotypes. Analysis of the phenotype reveals a strong bias towards right isomerism, indicative of defective left-sided identity specification during development. This is supported by gene expression data revealing weak or absent *Nodal* node expression and loss of downstream gene expression in the LPM. We use *in vitro* assays to demonstrate that ZIC2 can activate transcription from *Nodal* enhancer reporters. Our data suggest that ZIC2 acts upstream of *Nodal* expression at the node, possibly to prime the gene locus for the subsequent activation of its expression there. Together with a previous study that identified a role for ZIC2 in ciliogenesis, our results suggest a model in which ZIC2 acts at multiple levels during the establishment of laterality, upstream of genes and events that are critical for the process to take place.

## Results

### Cardiovascular and pulmonary malformations in human ZIC2 HPE cases suggest a laterality defect

Extra-craniofacial defects, including cardiovascular, visceral and urino-genital anomalies have previously been noted in patients with holoprosencephaly (HPE) carrying *ZIC2* mutations^2,3^, but the details of these malformations have not previously been documented. We examined clinical reports derived from a previously published European series consisting of 645 HPE probands^3^, including both liveborns and medically terminated pregnancies. A total of 67 probands in this series have *ZIC2* mutation, of which 8 (12%) exhibit cardiovascular or visceral anomalies suggesting a putative laterality defect (Table 1). The affected probands have mutations including three single amino acid substitutions (p.(His156Tyr); p.(Gln36Pro); p.(Phe314Cys); all affect highly conserved residues and are not found in 61,000 control exomes from the ExAC Project), alanine tract deletions and duplications, and larger chromosomal aberrations (Table 1; Fig. S1a,b).

**Table 1:** Association of cardiovascular and *situs* defects with holoprosencephaly in human cases with *ZIC2* mutation.

Proband	Gender	Survival	Mutation		Isolated/Syndromic	Type of HPE^c^	Heart malformations	Other visceral anomalies
			DNA^a^	Protein				
1	F	Fœtus MTP^b^	107 A > C	p.(Gln36Pro)	I	Semilobar	N/A	Renal hypoplasia, Pulmonary hypoplasia, Adrenal hypoplasia, Spleen hypoplasia
2	F	Fœtus MTP	466 C > T	p.(His156Tyr)	S	Semilobar	Hypoplastic ascendant aorta, VSD	Single umbilical artery
3	F	Fœtus MTP	941 T > G	p.(Phe314Cys)	S	Pseudo-anencephaly	Hypoplastic ascendant aorta, Aortic stenosis, VSD	Diaphragmatic hernia, Unilateral renal agenesis, Unicorn uterus
4	F	Fœtus MTP	1377–1406 del	Alanine tract deletion (aa456–465)	S	Lobar	N/A	Pulmonary hypoplasia Abnormal lung lobulation, Common mesentery, Single umbilical artery
5	F	Fœtus MTP	1377–1406 dup	Alanine tract expansion (increased to 25 from 15)	I	Semilobar	Subaortic VSD	Single umbilical artery
6	M	Fœtus MTP	1493 delG	p. (Gly498A fs*57)^d^	S	Semilobar	VSD	
7	M	Fœtus MTP	13q deletion	N/A	S	Alobar	Duplicated superior vena cava Tetralogy of Fallot Ductus arteriosus agenesis	Bilobulated lung Gallbladder agenesis
8	M	Born alive	Whole gene deletion	N/A	I	Syntelencephaly	Heart defect (not documented)	

Notes

^a^DNA co-ordinates are shown for the cDNA sequence starting from the beginning of the coding sequence.

^b^MTP = medical termination of pregnancy.

^c^Severity of HPE phenotype: Alobar > Semilobar > Lobar > Syntelencephaly.

^d^A single base deletion leads to a frameshift error resulting in translation of a false sequence with a stop codon after 57 amino acids.

Proband 7 has the most severe alobar form of HPE and also exhibits the most pronounced laterality defect (Table 1). This proband exhibits a loss of normal asymmetric thoracic anatomy, indicated by a duplicated superior vena cava (SVC) and a bilobulated lung. The right and left brachiocephalic veins are normally fused in man to form a single SVC which enters only the right atrium. Duplicated SVC therefore indicates bilateral connection to both atria, indicative of the laterality defect right atrial isomerism. Pulmonary morphology is also normally asymmetric in man such that, while the right lung has three lobes the left has only two. This patient has a symmetrical bilateral left-sided anatomy, suggesting left pulmonary isomerism. Thus, there is discordance between cardiovascular and pulmonary *situs* indicating *situs ambiguus*, a common phenomenon in heterotaxy.

Proband 4 also has abnormal lung lobulation indicating a laterality defect, but unfortunately the attending clinician did not record the details of this malformation and we are unable to assign *situs*. The same proband has additional features indicative of a laterality defect including a single umbilical artery (also seen in Probands 2 and 5) and a common mesentery. Proband 1 has spleen hypoplasia suggestive of right isomerism (which is also known as asplenia). This proband also has both adrenal and renal hypoplasia, features suggestive of abnormal abdominal *situs*. Pulmonary hypoplasia in this proband may indicate abnormal pulmonary *situs*.

Ventricular septal defect (VSD) is the most common cardiovascular anomaly observed (5 of 67 *ZIC2* probands). This is associated with hypoplastic ascending aorta in two probands, while another exhibits Tetralogy of Fallot. VSD is frequent in mouse models with a laterality defect^32^ but is not diagnostic because it is also commonly associated with other genetic conditions, such as Chromosome 22 deletions^33,34^.

In summary, this analysis indicates that HPE is seen in *ZIC2* probands together with cardiovascular, pulmonary and visceral malformations suggestive of an underlying laterality defect.

### Identification of the *Zic2* iso mutant

We performed a recessive ENU mouse mutagenesis screen in which MRI screening was used to identify novel mutants exhibiting cardiovascular anomalies at E14.5^35,36^. This resulted in isolation of the *iso* (isomeric) line. Mapping and sequencing revealed a stop-gain mutation (Y401X) in the *Zic2* gene disrupting the fifth zinc finger domain of the protein (Fig. S2; Table S1). The phenotype was validated through generation of a series of additional alleles, each with mutation targeted to the fifth zinc finger domain using TALEN-based gene editing^31^. Line *Zic2*^*A8*^ encodes an identical protein to *iso* (Y401X), while *Zic2*^*A5*^*, Zic2*^*A10*^*, Zic2*^*A17*^ and *Zic2*^*A19*^ have short DNA deletions leading to a frameshift followed by premature termination (*Zic2*^*A5*^*, Zic2*^*A10*^*, Zic2*^*A17*^) or an internal deletion (*Zic2*^*A19*^). All mutations affect the fifth zinc finger domain (Fig. S2) and thus differ from the previously published *Zic2*^*ku*^ mutant in which the fourth zinc finger is disrupted (C370S)^37^. *iso* fails to complement either *Zic2*^*A8*^ or *Zic2*^*A5*^ (Table 2).

**Table 2:** Neural tube, visceral and cardiovascular defects identified in mouse *Zic2* mutants.

			*iso*	TALEN			Trans-hets	
			iso/iso	*A5/A5*	*A10/A10 & A17/A17*	*A19/A19*	*iso/A5*	*iso/A8*
Neural Tube Defects	Exencephaly		17/17	10/10	3/3	3/3	2/2	2/2
	Holoprosencephaly	Alobar	13/16	5/7	3/3	2/3	2/2	2/2
		Semilobar	3/16	2/7	0/3	1/3	0/2	0/2
		Lobar	0/16	0/7	0/3	0/3	0/2	0/2
	Eyes	Cyclopia	8/17	3/7	1/3	2/3	1/2	2/2
		Hypotelorism	6/17	3/7	1/3	0/3	0/2	0/2
		Absent	3/17	0/7	1/3	1/3	1/2	0/2
	Spina bifida		17/17	10/10	3/3	3/3	2/2	2/2
	Curly tail		17/17	10/10	3/3	3/3	2/2	2/2
Viscera	Lungs	Right Isomerism	13/17	6/10	2/3	3/3	1/2	2/2
		Left Isomerism	0/17	1/10	0/3	0/3	0/2	0/2
		Reversed	0/17	0/10	0/3	0/3	0/2	0/2
		Normal	4/17	3/10	1/3	0/3	1/2	0/2
	Spleen	Reduced or absent	1/6	4/7	0/2	0/0	0/1	0/0
		Duplicated	0/6	0/7	0/2	0/0	0/1	0/0
		Right sided	2/6	0/7	0/2	0/0	0/1	0/0
	Heart	Dextrocardia	4/16	4/10	0/3	2/3	0/2	1/2
		Mesocardia	7/16	2/10	0/3	1/3	2/2	1/2
	Stomach	Right sided	7/17	2/10	0/3	0/3	0/2	2/2
	Pancreas	Right sided	5/14	2/7	0/3	0/2	0/2	0/0
Atria & Veins	Bilateral systemic venous sinus		10/15	4/10	1/3	3/3	1/2	2/2
	Left sided Inferior vena cava		5/16	3/10	0/3	1/3	0/2	2/2
	Hepatic vein drainage direct to atria		2/16	3/10	0/3	3/3	0/2	0/2
	Atrial septal defect		8/11	5/10	1/3	2/2	1/2	2/2
Ventricles & Arteries	Abnormal ventricular topology		6/13	6/10	0/3	1/2	1/2	2/2
	Great artery transposition		0/13	0/10	1/3	0/3	0/2	0/2
	Double outlet right ventricle		5/13	5/9	0/3	2/3	0/2	2/2
	Ventricular septal defect		11/15	7/10	0/3	1/1	2/2	2/2
	Rightward looped aortic arch		5/17	3/10	1/3	2/3	0/2	0/2

A summary of the phenotypes observed by MRI and µCT imaging in all *Zic2* mutants examined including both the original *iso* line and TALEN-generated lines. All embryos are homozygous mutants, except for those labelled “trans-hets” which carry one *iso* allele and one TALEN allele as a test of complementarity. For each phenotype described, the first number indicates the number of embryos observed with that phenotype, while the second indicates the number examined. The latter number differs for different phenotypes because it was not possible to assess every phenotype in every embryo due to limitations of imaging.

### *Zic2* mutants exhibit holoprosencephaly

**Figure 1:**
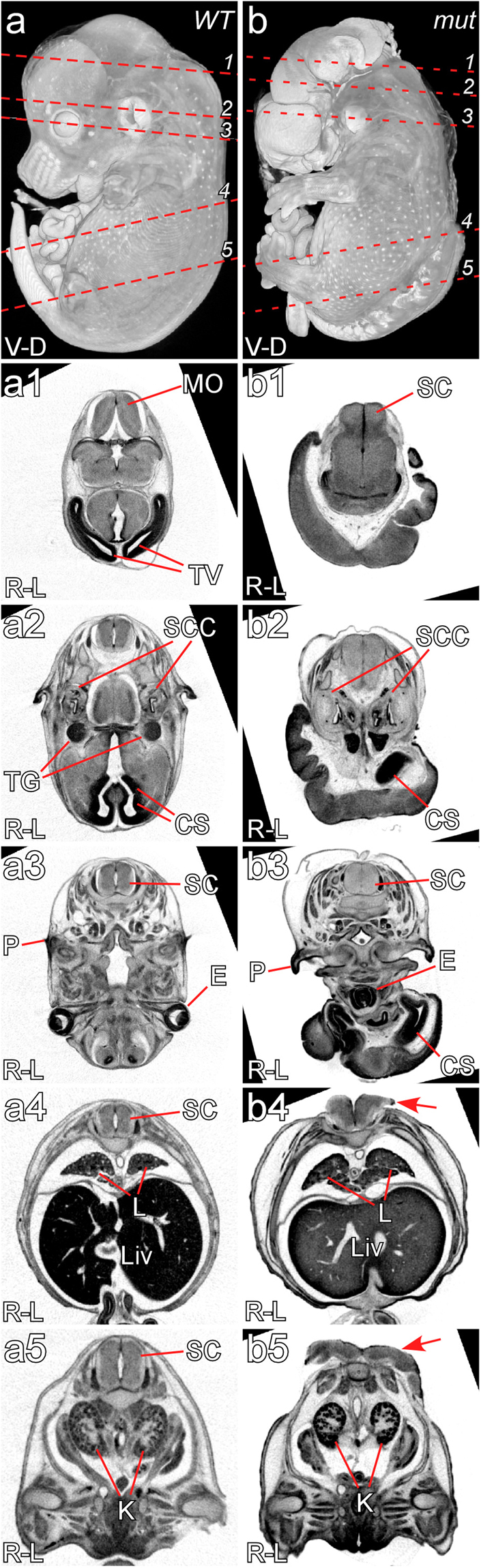
Holoprosencephaly, spina bifida and cyclopia in the Zic2 mouse mutant. Representative images illustrating a wildtype E14.5 embryo (**a**,a1–a5) and a *Zic2*^*A5/A5*^ embryo (**b**,b1–b5) at E14.5. Dashed red lines in a and b indicate the level of sections shown below. The mutant embryo exhibits alobar holoprosencephaly indicated by a lack of separation in forebrain structures (b1,b2), cyclopia (b3), spina bifida (b4,b5 arrows) and right pulmonary isomerism (b4). Abbreviations: V-D – ventral – dorsal; R-L – right – left; MO – medulla oblongata; SC – spinal cord; SCC – semi-circular canal; TG – trigeminal ganglion; TV – telencephalic vesicle; CS- corpus striatum; P – pinna; E – eye(s); L – lungs; Liv – liver; K – kidneys.

**Figure 2:**
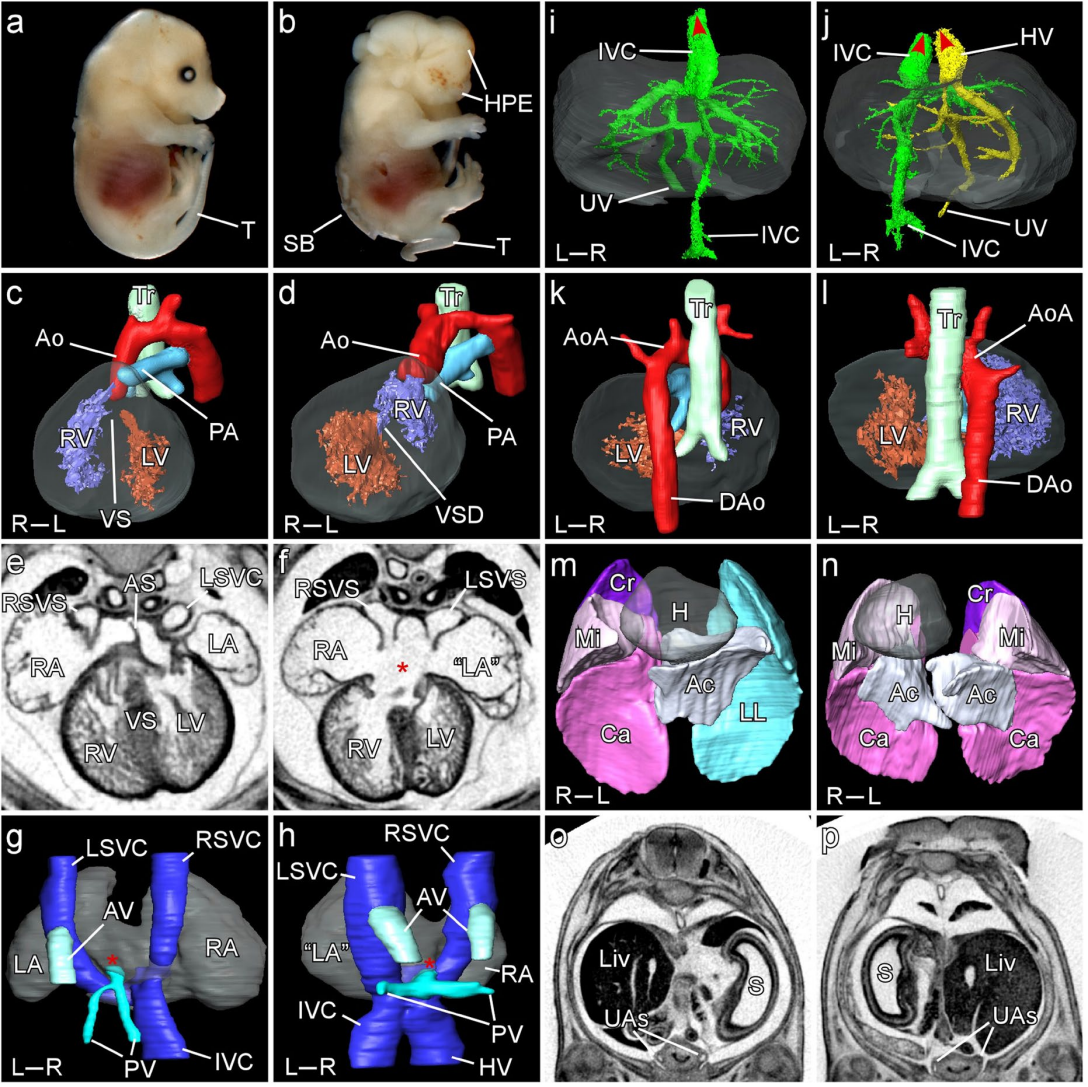
Cardiovascular, pulmonary and visceral phenotypes in the Zic2 mouse mutant. Representative images illustrating range of phenotypes observed in wildtype (**a**,**c**,**e**,**g**,**i**,**k**,**m**,**o**) and homozygous mutants (*iso* and TALEN; **b**,**d**,**f**,**h**,**j**,**l**,**n**,**p**) at E14.5. (**a**,**b**) Holoprosencephaly (HPE), *spina bifida* (SB) and curly tail (T). (**c**,**d**) Reversed ventricular topology. The left ventricle (LV) is dextral to the right (RV), which gives rise to both Ao and PA (double-outlet right ventricle). (**e**,**f**) Right atrial isomerism indicated by bilateral systemic venous sinuses (SVS) and a large atrio-ventricular septal defect (asterisk). (**g**,**h**) Systemic and pulmonary veins (dorsal view). In the control embryo, the great veins (Inferior vena cava (IVC), left superior vena cava (LSVC), right superior vena cava (RSVC)) converge to form the systemic venous sinus which drains into the right atrium (RA) while the pulmonary veins (PV) drain into the left atrium (red asterisk). The azygos vein (AV) drains into the LSVC just above the level of the atria. In the mutant embryo, the IVC is left-sided while the hepatic vein (HV) and PV also drain into sinus venosus (red asterisk) which opens bilaterally into the atria. The AV is duplicated. (**i**,**j**) Hepatic venous anatomy (ventral view). In the wildtype, the IVC passes through the liver (grey shading) to drain into the right atrium (red arrowhead) and is connected to the umbilical vein (UV). In the mutant embryo, the IVC drains into the left atrium. The UV connects to the right hepatic vein (HV) which drains directly into the right atrium (red arrowheads). (**k**,**l**) Right-sided aortic arch (AoA). The descending aorta (Dao) may be seen to be ectopically located to the right of the trachea (Tr) in the mutant. (**m**,**n**) Right pulmonary isomerism. H – heart; LL – left lung; Cr – cranial lobe; Mi – middle lobe; Ca – caudal lobe; Ac – accessory lobe. (**o**,**p**) Ectopic right sided stomach (S). Liv – liver; S – stomach; UAs – umbilical arteries.

External examination revealed that all *Zic2* mutant embryos (n = 37) had neural tube defects including exencephaly, *spina bifida* and curly tail (Table 2; Figs 1; 2a,b); *spina bifida* was also present in 2/27 heterozygotes, which also frequently show curly tail (Fig. S3^31^), while wild-type embryos (n = 22) had no anomalies. We employed µCT and MRI imaging to analyse the phenotype more closely. All embryos examined had holoprosencephaly (Table 2; Fig. 1). A range of severity was seen, but the majority of embryos had the most severe alobar form in which the two hemispheres are completely fused (27 of 33; Fig. 1b2). The remaining 6 had the semilobar form, indicating partial hemisphere fusion. No embryos were observed to have the mild lobar form of HPE. Six of 33 embryos lacked eyes, cyclopia was seen in 17 of 33 embryos (Fig. 1b3). and hypotelorism in 10 of 33. These neural tube phenotypes are consistent with those of the previously described *Zic2*^*Ku*^ mutant^4,37^, suggesting that *Zic2*^*iso*^ may also carry a loss of function mutation.

### *Zic2*^*iso*^ mutants have extensive cardiovascular, thoracic and abdominal malformations

CT imaging also revealed extensive defects within the cardiovascular system, thorax and abdomen of *Zic2*^*iso*^ and TALEN mutants (Fig. 2; Table 2), many of which have not been previously described for *Zic2*.

We observed severe cardiovascular malformations in the majority of embryos. Abnormal ventricular topology was seen in half of all embryos examined (16 of 32; Table 2) such that the morphologically right ventricle was positioned on the left side of the embryo (Fig. 2c,d), while it was normal in the other 16. This 50–50 split indicates that the direction of heart tube looping was randomly assigned in these embryos, consistent with the phenotype previously described in younger *Zic2*^*Ku*^ embryos^24^. The majority of embryos had an *ostium primum* atrial or atrio-ventricular septal defect (19 of 30; Fig. 2e,f asterisk). 21 of 35 embryos exhibited bilateral systemic venous sinuses (Fig. 2e–h), often with bilateral right-sided atrial appendages. Double outlet right ventricle was seen in 14 of 32 embryos examined (Fig. 2c,d) and ventricular septal defect was present in 23 of 33 embryos (Fig. 2c,d). Defects were observed in the vascular system at reduced penetrance relative to the cardiac defects, and were generally present in about a third of embryos. The inferior vena cava (IVC) was found to be aberrantly left-sided in 11 of 36 embryos (Fig. 2g–j), while 8 of 36 exhibited hepatic vein drainage directly into the atrium, bypassing the IVC (Fig. 2g,h). A right-sided aortic arch was observed in 11 of 37 embryos (Fig. 2k,l).

In mouse, the right lung is divided into four distinct lobes while the left consists of a single lobe (Fig. 2m). We observed bilateral multilobed lungs in 27 of 37 mutant embryos (Figs 1b4; 2n), while a single embryo was observed with a 1:1 lobed arrangement (Table 2). All control and heterozygous embryos showed a normal 4:1 lobed arrangement. The stomach and pancreas are normally located on the left side of the body but were observed to be ectopically right sided in 11 of 37 and 7 of 28 mutant embryos respectively (Fig. 2p; Table 2). The spleen was reduced or absent in 5 of 16 embryos and right-sided in another two (Table 2).

### *Zic2*^*iso*^ mutants have right isomerism

A random distribution in the direction of looping of the heart tube and gut among mutant embryos is indicative of a generic laterality defect, but does not provide information on the specific *situs* of affected individuals. This may be assigned based on careful examination of the anatomy of the lungs and atria, the positioning of visceral organs and the organisation of the vascular system, all of which show precise phenotypes that may be classified as right-isomerism, left-isomerism or *situs inversus*^17,38^.

Pulmonary anatomy is a useful indicator of thoracic *situs* because the lungs have a lateralised morphology determined by *Pitx2c* expression. Bilateral multi-lobed lungs are indicative of a lack of *Pitx2c* expression (right isomerism) while bilateral unilobar lungs indicate bilateral *Pitx2c* expression (left isomerism). *Situs inversus*, in contrast (right-sided *Pitx2c* expression), would be expected to result in a four-lobed lung on the left side and a single lobed lung on the right. Our data indicate that 73% of *Zic2* embryos (27/37) exhibit right pulmonary isomerism, 3% (1/37) exhibit left isomerism, 24% (9/37) have the normal anatomy (*situs solitus*) and no embryos have reversed *situs*. This distribution is significantly different from a random distribution of the four phenotypes, and reveals a strong bias towards the phenotype of right isomerism (Chi squared test, p = 5.7 × 10^−11^). Similarly, bilateral systemic venous sinus and bilateral right atrial appendage is indicative of right atrial isomerism. We observe bilateral systemic venous sinus (right atrial isomerism) in 60% of embryos (21/35), with no evidence for left isomerism or reversed *situs*. This distribution is also significantly different from a random distribution (Chi squared test, p = 3.1 × 10^−8^). Double outlet right ventricle (44%; 14/32) is associated with right but not with left isomerism^32,39^. Abnormal right-sided aortic arch looping is not seen in left isomerism^40^ but is present in right isomerism^32,41^ and in *situs inversus*. We did not observe vascular phenotypes associated with left isomerism such as interruption of the inferior vena cava and partial anomalous pulmonary return^17^. Asplenia is commonly associated with right isomerism^42^, and indeed the human disease is sometimes known as Asplenia. The spleen is reduced in mice lacking the NODAL receptor *Acvr2b*, although asplenia seems to be observed only in *Cfc1* (*Cryptic*) mutants^41,43^. We observe reduced or right-sided spleen in 44% of embryos (7/16) while none exhibited polysplenia, a feature of left isomerism.

These data thus indicate a strong bias towards right isomerism. Only one embryo exhibits any feature of left isomerism (bilateral unilobed lungs) and this embryo does not show atrial left-isomerism nor any vascular phenotype associated with left-isomerism.

### The NODAL pathway is downregulated in *Zic2* mutants

**Figure 3:**
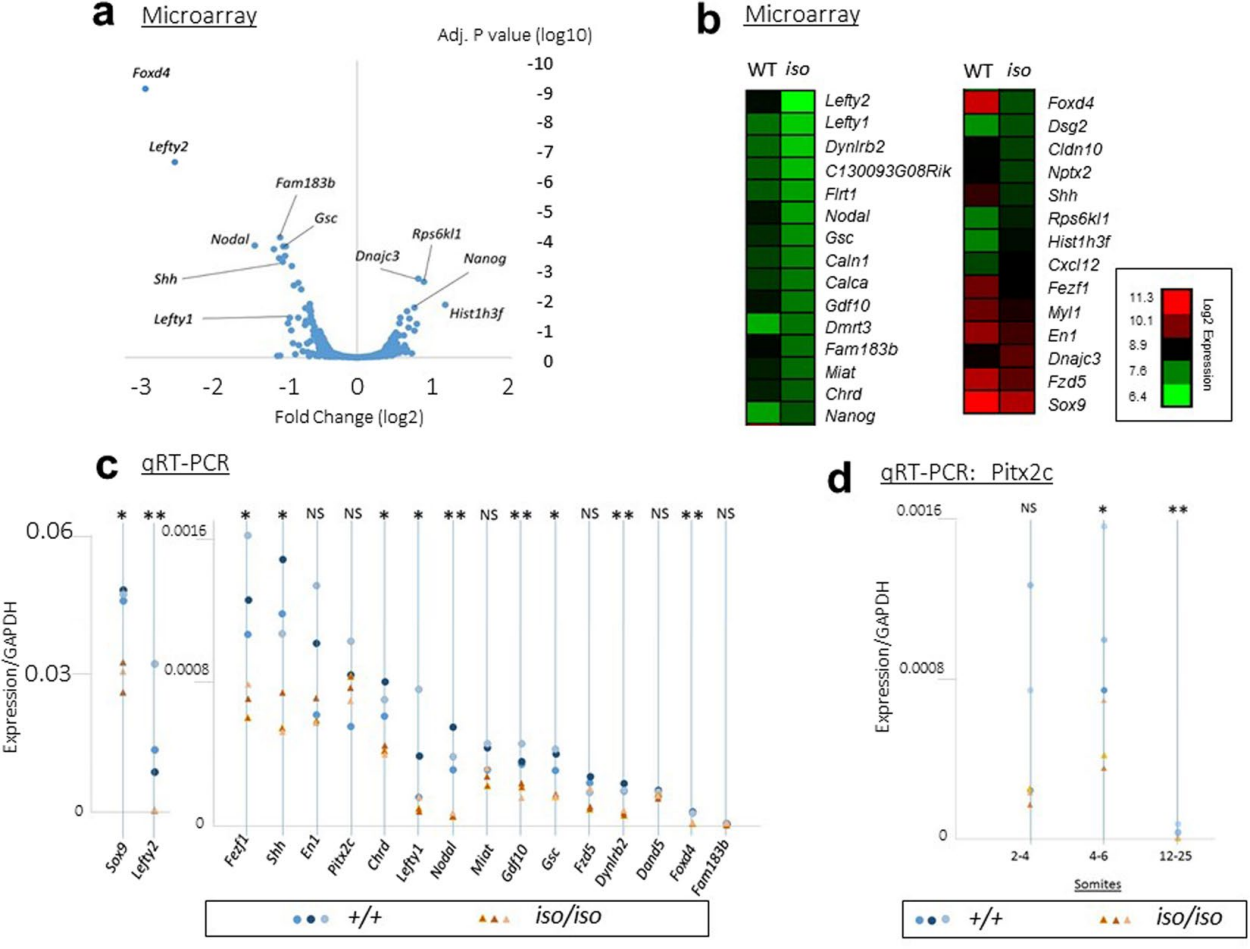
Loss of *Zic2* leads to downregulation of the NODAL pathway. (**a**,**b**) Microarray analysis of gene expression changes in the *Zic2*^*iso/iso*^ embryo compared to wildtype at E8.5. (**a**) Volcano plot. The data are plotted as log fold change (relative to wildtype) against adjusted p-value. (**b**) Heat map showing wildtype (WT; left panel) and *Zic2*^*iso/iso*^ (KO; right panel) embryos. Data show mean expression for three independent biological replicates of pooled embryos and are expressed as log2 values. (**c**) SYBR Green based qRT-PCR analysis of genes identified in the microarray screen, and known NODAL pathway genes. Expression values shown are normalised to *Gapdh*. Three independent biological replicates of pooled embryos (0–4 somites) were performed for each condition and these are plotted as individual data points (blue circles indicate wildtype; orange triangles indicate *Zic2*^*iso/iso*^). Asterisks above each column indicate result of a one tailed t-test of samples with unequal variance testing the null hypothesis that loss of *Zic2* has no effect on gene expression: ***p < 0.0005; **p < 0.005; *p < 0.05, NS = not significant. (**d**) Taqman qRT-PCR analysis of Pitx2c expression at three different ages (2–4 somites, 4–6 somites and 12–25 somites). Three independent biological replicates of pooled embryos were performed for each condition. Labels as per panel c.

We performed a microarray experiment in mice as an unbiased screen to identify putative *Zic2* transcriptional targets. Global gene expression was assayed in whole *Zic2*^*iso/iso*^ embryos harvested at E8.0 -E8.5 (0–4 somites) relative to wild-type. Only 29 genes showed a significant change in their expression level (with a false discovery rate of 5%) and the majority (23/29) were downregulated (Fig. 3a,b; Table S2). Some of these, such as *Dmrt3* and *Fzd5*, are specifically expressed in the developing head. Other downregulated genes have an established role in left-right patterning including *Lefty2* (−5.1), *Nodal* (−2.5), *Lefty1* (−1.8) and *Shh* (−2.0). Downregulated genes also include *Gdf10* (−2.01) and *Chordin* (−1.53), which are, like *Nodal* and *Lefty*, associated with TGFβ signalling, and *Sox9* which has not so far been associated with LR establishment in the mouse although it is known to be involved in sea urchin^44^. Many of the changed genes, including *Nodal, Shh, Fam183b, Foxd4* and *Dynlrb2* are known to be expressed within the node, while others including *Gsc* and *Lefty1* are expressed in node-derived cells at the midline. Upregulated genes included the pluripotency factor *Nanog* (+1.67). To validate these changes we performed quantitative real-time polymerase chain reaction (qPCR) assays for changed genes, as well as for the NODAL pathway genes *Pitx2c* and *Dand5* (also known as *Cerl2*; Fig. 3c). This analysis confirmed the downregulation of *Nodal, Lefty2, Lefty1 and Shh*. Surprisingly, *Pitx2c* was not changed. We hypothesised that this might be because the assay was performed at an age before *Pitx2c* is upregulated in the LPM, and therefore repeated the analysis using older embryos. In older embryos (aged 4–6 somites), we observed a significant reduction in expression, which persists in embryos of 12–25 somites (Fig. 3d), indicating that *Pitx2c* is downregulated in the *iso* embryo during the time at which it is normally expressed in the LPM. Thus, these data suggest downregulation of the NODAL pathway in the *iso* embryo.

### *Zic2* is required for Nodal expression in perinodal crown cells

**Figure 4:**
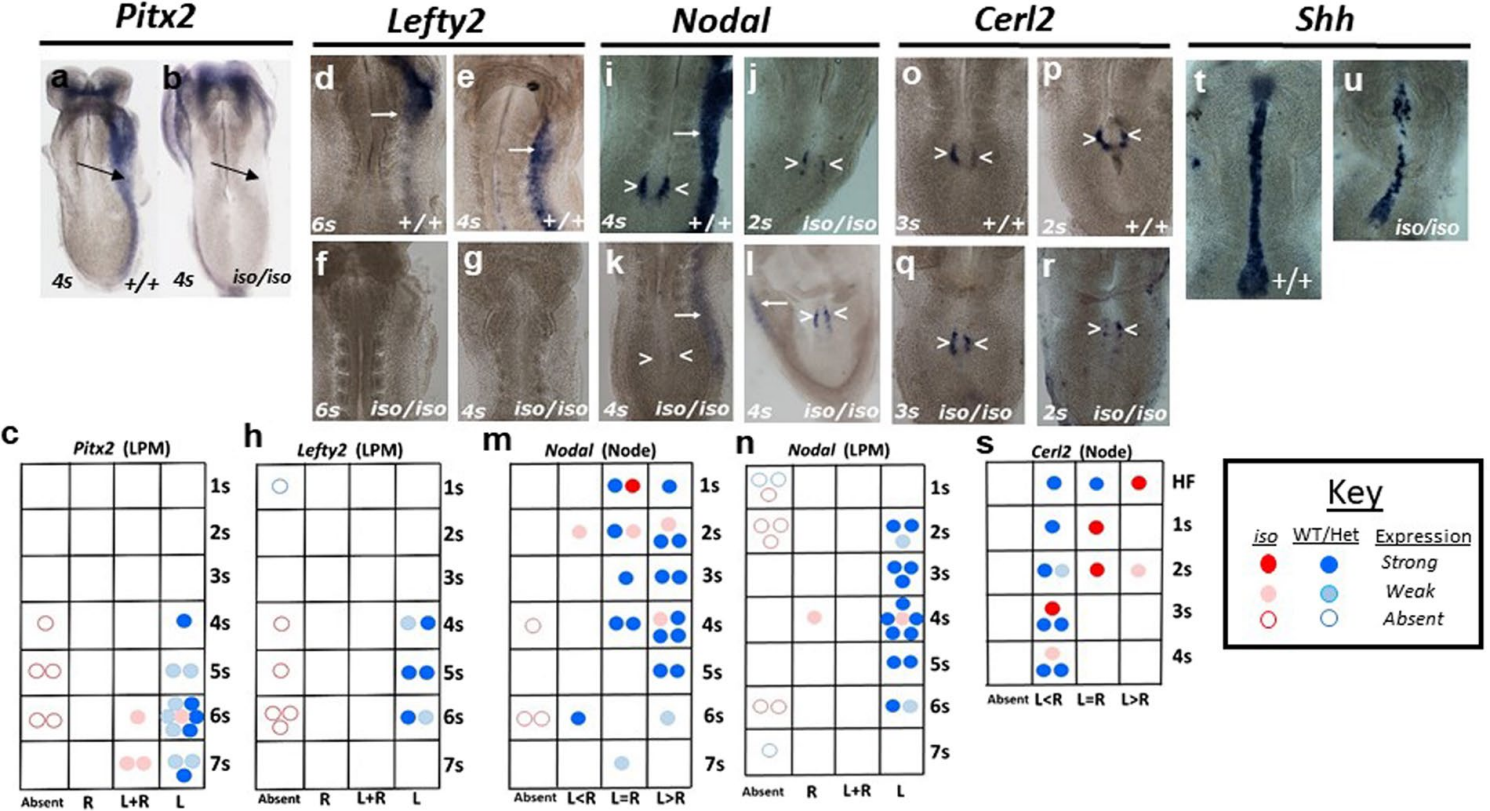
Disrupted left-side specification in the *Zic2* mutant. **(a**,**b**; **d**–**g**; **i**–**l**; **o**–**r**; **t**,**u**) *in situ* hybridisation. Genotype and age of embryos (expressed as somite number) are indicated. All images are oriented with the left side on the right of the photo and show the ventral surface of the embryo. Arrows indicate LPM, arrowheads: node. t and u are composite images. (**c**,**h**,**m**,**n**,**s**) Charts summarise the results of *in situ* experiments illustrating the relationship of expression pattern and expression level to embryo age and genotype. Each dot represents an individual embryo, blue dots are control embryos (+/+ and +/*iso*), red are mutants (*iso/iso*). A dark colour indicates strong expression while a lighter colour indicates weak or absent expression. Data are plotted as expression pattern (one of 4 categories shown on the *x* axis) against embryo age (expressed as somite number: 1 s, 2 s etc., *y* axis). HF = headfold stage. L = left, R = right.

We used *in situ* hybridisation to further analyse changes in the core NODAL pathway genes. *Pitx2* expression was investigated using a probe which recognises all three *Pitx2* isoforms^45^ and was found to be expressed in the head folds and in the LPM in the wildtype embryo (Fig. 4a). In *Zic2*^*iso/iso*^ embryos *Pitx2* expression was seen in the head folds, but was weak or, in many cases, absent from the LPM (Fig. 4b, arrow). Expression appeared to be delayed from the 4 to the 6-somite stage, it was weaker, and its laterality was perturbed, often bilateral (Fig. 4c). *Lefty2* was expressed exclusively in the left LPM of wildtype embryos aged between 4 to 6 somites (Fig. 4d,e). All *Zic2*^*iso/iso*^ embryos failed to express *Lefty2* (Fig. 4f–h). *Nodal* was expressed in the node of all wildtype embryos examined (Fig. 4i, arrowhead) and was either bilaterally expressed or was enriched on the left side of the node (Fig. 4m). In the LPM, *Nodal* was expressed in all wildtype embryos from 2 to 6 somites and was restricted to the left side (Fig. 4i, arrow; Fig. 4n). *Nodal* expression at the node of *Zic2*^*iso/iso*^ embryos was in most cases (7 of 8) weak or absent (Fig. 4j–m). Expression was only detectable in early somite stages (1 to 4 somites) mutant embryos (5 of 8), and its abnormal laterality in one of them suggested a defective nodal flow, which would be consistent with the requirement for *Zic2* in node cilia development or function as previously proposed^24^. Most *Zic2*^*iso/iso*^ embryos (6 of 8) failed to express *Nodal* in the left LPM (Fig. 4j,n). In the two mutant embryos that did express *Nodal* in the LPM, it was weak and was observed in both left (Fig. 4k) and right LPM (Fig. 4l). *Cerl2 (Dand5)*, known to be the earliest asymmetrically biased gene expressed at the node, (L < R)^46^ was expressed exclusively within the node of wildtype embryos (Fig. 4o,p) and was observed to be enriched on the right side of the node in 8 of 9 embryos (Fig. 4s). *Cerl2* appeared to be maintained at wildtype levels in *Zic2*^*iso/iso*^ embryos (Fig. 4q–s), but the onset of its laterality appeared to be delayed and slightly perturbed (Fig. 4s), an observation again consistent with the possibility that the mutation of *Zic2* initially results in defective nodal flow and randomisation of gene expression at the node. We also studied the expression of *Shh*, which was detected along the embryonic midline in wildtype embryos in a solid band (Fig. 4t). In *Zic2*^*iso/iso*^ embryos, expression levels were maintained but there seemed to be fewer positive cells and the band of expression appeared disrupted (Fig. 4u) suggestive of impaired development of *Shh*-expressing cells in these embryos.

In summary, we find some anomalies suggestive of an occasional randomisation of LPM identity (bilateral *Pitx2c* expression, right-sided expression of *Nodal*). These anomalies match the laterality defects we characterized in the expression of *Nodal* and *Cerl2* at the node, defects which are consistent with the *Zic2* mutation leading to nodal flow perturbations, most likely as a result from the node cilia defect described in *Zic2*^*Ku*^^24^. However, our data show that the predominant phenotype is the absence of expression in the LPM. This failure to activate NODAL-dependent gene expression (*Nodal, Pitx2c, Lefty2*) in the LPM is consistent with the later bias towards right-isomerism revealed by our analysis of the *Zic2*^*iso*^ mutant phenotype. *Nodal* expression within the node, a prerequisite for downstream gene expression in the LPM^13,46^, is reduced or absent in most embryos. These data suggest that ZIC2 may be required for *Nodal* expression at the node.

### ZIC2 binds to the Nodal locus and can activate transcription

**Figure 5:**
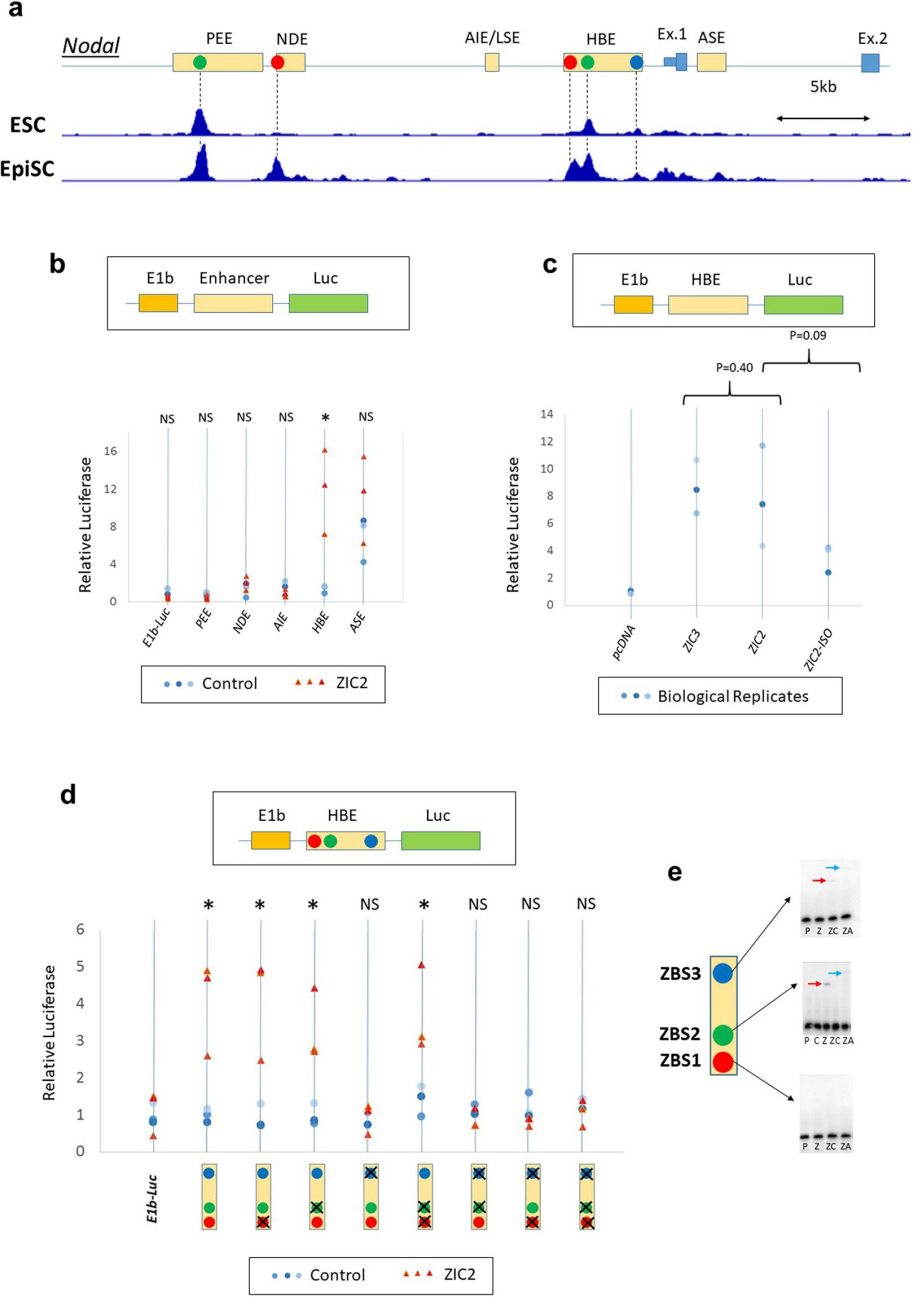
ZIC2 binds to the *Nodal* locus and promotes transcriptional activation. (**a**) ChIP-seq data to show ZIC2 binding at the *Nodal* locus in embryonic stem cells (ESC)^27^ and in epiblast stem cells (EpiSC)^26^. The five previously-characterised enhancer elements are indicated by yellow boxes in the cartoon above, while exons are indicated by blue boxes. Coloured dots in the cartoon indicate putative ZIC2 binding sites. In ESCs (upper trace), two high-affinity binding sites are seen, located within the PEE and HBE enhancers (green dots). In EpiSCs (lower trace), four high-affinity sites are observed which includes the two bound in ESCs (green dots) as well as two additional sites located within the NDE and HBE enhancers (red dots). Several lower-affinity binding sites map to ASE, HBE and exon 1, the site located within HBE is indicated by a blue dot. (**b**) Luciferase assays performed using reporter constructs consisting of each of the known *Nodal* enhancers linked to a viral E1b promoter and luciferase, as shown in the cartoon. Blue circles indicate control transfections, orange triangles ZIC2 transfections. Luciferase activity is plotted relative to control. The data show that the HBE reporter is activated by ZIC2 above background. (**c**) Luciferase assays using the native HBE reporter, performed as in b. Cells were transfected with the reporter and an expression plasmid for ZIC3, ZIC2 or ZIC2-ISO, or a control empty vector (pcDNA). (**d**) Luciferase assays using modified HBE reporters, performed as in b. HBE contains 3 putative ZIC2 binding sites, indicated by the coloured dots in the cartoon, deleted binding sites are indicated by an “X”. Deletion of sites ZBS1 and ZBS2 (red and green dots in the cartoon) has no effect on the ability of ZIC2 to activate the reporter, while deletion of site ZBS3 (blue dot) eliminates this activity. (**e**) Gel shift (EMSA) assays to study the binding of ZIC2 to HBE. P = probe only, Z = probe + HA-ZIC2, ZC = probe + HA-ZIC2 + unlabelled competitor, ZA = probe + HA-ZIC2 + αHA antibody. Red arrow = gel shift, blue arrow = supershift. Images show cropped gel pictures, uncropped images are shown in the supplemental data.

The expression of *Zic2* in the epiblast overlaps with that of *Nodal* from the blastocyst stage to the late gastrula stage, up to and including formation of the node^6,7^. The dynamic expression of *Nodal* during development is regulated by five enhancers^47–49^. Analysis of previously published ChIP-seq datasets^26,27^ shows that ZIC2 is bound to the *Nodal* locus in both ESCs and in EpiSCs (Fig. 5a). In ESCs, prominent binding peaks were mapped to the Proximal Epiblast Enhancer (PEE)^50^ and to the Highly Bound Element (HBE)^49^ (Fig. 5a, green dots). These binding sites are also occupied in EpiSCs, but two additional high-affinity binding peaks are seen within the Node Dependent Enhancer (NDE)^51^ and in HBE (Fig. 5a, red dots) indicating that ZIC2 binding is more widespread in these cells. Further low-affinity peaks map to the Asymmetric Enhancer (ASE)^48,51^, to Exon 1 and to HBE (blue dot).

We tested the ability of ZIC2 to regulate the transcriptional activity of each of the five *Nodal* enhancers using luciferase reporter constructs in which the enhancer is linked to the minimal E1b promoter^49^ (Fig. 5b). Each construct was co-transfected in U2-OS cells together with an expression plasmid encoding ZIC2 or an empty vector (pcDNA).

The ASE enhancer showed a strong basal activity in control cells suggesting activation by endogenous factors present in U2-OS cells (Fig. 5b). For this reason, we were not able to accurately test the ability of ZIC2 to activate this enhancer. Low basal activity was observed for the remaining enhancers. We found no evidence for ZIC2-mediated transcriptional activation from the PEE, NDE or AIE enhancers. HBE, in contrast, shows a robust ZIC2-mediated transcriptional activation. HBE has been shown to be transcriptionally active in primitive streak-like cells (node precursors)^49^.

*Zic3* has a phenotype closely resembling that of *Zic2*, including laterality-related malformations and HPE^52,53^, and also exhibits reduced or absent expression of *Nodal*^52^. ZIC3 has previously been shown to be able to activate an NDE enhancer^54^. We therefore asked whether ZIC3 can also activate expression from the HBE enhancer. Luciferase assays demonstrate that ZIC3 activates HBE at a level similar to that of ZIC2 (Fig. 5c), lending further support to the hypothesis that this enhancer may be important in LR patterning.

We next asked whether the *iso* mutation reduces the ability of ZIC2 to activate the HBE enhancer, and thus whether a failure to activate *Nodal* might explain the phenotype. The *Zic2 kumba* allele has been shown to evade nonsense mediated decay (NMD) but to produce a protein which cannot activate the *ApoE* promoter in luciferase assays^55^. While the *kumba* mutation disrupts a cysteine residue in the fourth zinc finger^37^, the *iso* mouse carries a Y401X mutation which disrupts the fifth zinc finger of ZIC2 (Fig. S1). Two lines of evidence indicate that *iso*, like *kumba*, may evade NMD. Firstly, *Zic2* mRNA is not significantly reduced in *iso* embryos by microarray analysis (Table S2). Secondly, qRT-PCR analysis indicates that the mRNA can be detected in mutant embryos (Fig. S4). We used site-directed mutagenesis to generate a Y401X mutation within the ZIC2 expression construct (named ZIC2-ISO) and tested the response of the HBE luciferase reporter to this. ZIC2-ISO shows a reduced ability to activate HBE (Fig. 5c), just above the threshold of significance (p = 0.09), but still maintains some transcriptional activity. This, together with potential redundancy with ZIC3, may explain the partial penetrance of the Zic2^iso^ phenotype.

To gain further insights into the regulation of HBE by ZIC2, we investigated ZIC2 binding sites within HBE. ChIP-seq data suggests the presence of three putative binding sites, and we found that each of these contains sequence matching the consensus ZIC2 binding motif. ZIC2 Binding Site 1 (ZBS1) is located at position 99–109 bp within HBE and has the sequence CACCTCCTGGG (Fig. 5a,d,e red dot), ZBS2 at position 615–625 bp with sequence CCCCTGGGGTG (Fig. 5a,d,e green dot), and ZBS3 at 1845–1855 bp with sequence GCCCTCCTGGG (Fig. 5a,d,e blue dot). We used site-directed mutagenesis to delete each of these sites within the HBE-luciferase reporter construct. Luciferase assays indicated that while deletion of sites ZBS1 and ZBS2 has no effect on ZIC2-mediated transcriptional activation, reporters lacking ZBS3 completely lost their responsiveness to ZIC2, indicating that an intact site ZBS3 is an absolute requirement (Fig. 5d).

An electrophoretic mobility shift assay (EMSA) confirmed binding of ZIC2 to ZBS2 (occupied in both ESCs and EpiSCs) and ZBS3 (required for activation but demonstrating only low-affinity binding in ESCs and EpiSCs) but not to ZBS1 (occupied in EpiSCs but not in ESCs) *in vitro* (Fig. 5e).

Thus, these data demonstrate that the HBE enhancer, which is bound by ZIC2, both *in vivo* in node precursors (Fig. 5a) and *in vitro* (Fig. 5e), can mediate ZIC2-dependent and ZIC3-dependent transcriptional activation *in vitro* (Fig. 5b,c). An identified ZIC2 site within HBE, bound at low-affinity in precursor cells (Fig. 5a) is required for this activity *in vitro* (Fig. 5d) and a ZIC2 mutation reproducing the *iso* mouse mutant, which has impaired *Nodal* node expression (Fig. 4j–m), can reduce the ability of ZIC2 to activate HBE (Fig. 5c).

## Discussion

In this work, we reveal that *ZIC2*/*Zic2* loss in both man and mouse is associated with a complex set of previously unappreciated malformations affecting the cardiovascular, pulmonary and digestive systems, in addition to the better known neural tube defects. The mouse phenotype reveals a laterality defect which shows a strong bias towards right-isomerism, indicative of a lack of left-sided identity in the lateral plate mesoderm during morphogenesis of these organ systems. This is supported by molecular data indicating that the left-determining NODAL signalling cascade fails to be activated in the LPM. *Nodal* expression at the node is also impaired, implying that ZIC2 acts upstream of this event. Binding of ZIC2 to the *Nodal* locus in EpiSCs, together with *in vitro* data revealing ZIC2-mediated transcriptional activation of regulatory sequences, suggests that a direct interaction between ZIC2 and *Nodal* is critical for the establishment of left-sided mesoderm identity.

The association of *Zic2* with cardiovascular laterality defects was first demonstrated in the *Zic2*^*Ku*^ mouse, in which the direction of heart tube looping was shown to be randomly assigned^24^. Node cilia were found to be reduced in length in these mutants from 4 µm to 2.5 µm, and this, together with the earlier observation that *Zic2* expression in the node is turned off just before *Nodal* expression in perinodal cells is initiated^6^, led to the conclusion that the laterality defect (interpreted as random cardiac *situs*) resulted from impaired nodal cilia development^24^. A randomised distribution in the direction of heart tube looping within a population of mutants does not in itself indicate randomised cardiac *situs*. Such a phenotype is also associated with specific, non-random laterality defects such as isomerism^32,56^. This is because, in the absence of laterality, heart looping lacks directionality and may turn in either direction. The *Zic2*^*Ku*^ mutant exhibits mid-gestation lethality, preventing further investigation. In contrast, most *Zic2*^*iso*^ and TALEN mutants survive until E15.5, which has allowed us to perform a more detailed analysis of the laterality phenotype. We confirmed the heart tube looping phenotype but revealed that this results not from randomisation of *situs*, but from right isomerism. *Zic2*^*iso*^ and TALEN mutants show a strong bias towards right isomerism over other laterality phenotypes. Pulmonary *situs* was assessed in a total of 37 *Zic2* mutant embryos, of these 73% showed right pulmonary isomerism, a single embryo exhibited left isomerism and none had *situs inversus*. The same phenotype was seen in multiple independent *Zic2* alleles. Laterality of the cardiovascular system matched that of the thorax, albeit at reduced penetrance, as demonstrated, for example, in right atrial isomerism, which was seen in 60% of embryos. Many other features indicate right isomerism and not left isomerism or *situs inversus*.

This conclusion is consistent with the observed molecular phenotype. Both *Zic2*^*Ku*^ and *Zic2*^*iso*^ mutants fail to activate NODAL signalling in the LPM. *Zic2*^*Ku*^ mutants lack *Nodal* and *Lefty2* LPM expression, and have reduced *Pitx2* expression^24^. Our analysis reveals a similar molecular phenotype in the *Zic2*^*iso*^ mutant. Mutations which impair the motility of node cilia such as the *iv* mutant of the cilia dynein *Dnah1*^12^, or mutations in transcription factors required for cilia development such as *Noto*^57^, result in the stochastic activation of NODAL signalling in the LPM. This is visualised via detection of the expression of *Nodal/Pitx2c/Lefty2* with the same frequency on the left, on the right, on both sides or in none, and leads at later stages to equal proportions of embryos with *situs solitus, situs inversus*, right isomerism or left isomerism. Only 4 out of 20 E8.5 *Zic2*^*iso*^ mutant embryos examined showed ectopic expression of these genes in the LPM. 15 failed entirely to express them in this tissue, an observation consistent with the fact that 73% of those examined at E14.5 exhibited right isomerism. These numbers do not support the hypothesis that a defect in cilia motility is the major cause of the laterality defect.

Although there is ample evidence that cilia motility conditions the asymmetry of *Nodal* expression in perinodal cells, this asymmetry in mRNA expression is not itself required to induce *Nodal* expression in the left LPM^47,58,59^. What appears to matter is the amount of NODAL protein produced. Pioneering studies relying on the removal of specific *Nodal* enhancers have established that *Nodal* expression at the node is required to induce *Nodal* expression in the LPM^13,58,59^ thus providing an explanation for the right isomerism of *Zic2* mutant embryos that fail to express *Nodal* at the node. However these studies also showed that residual *Nodal* expression at the node could be sufficient to induce correct expression in the LPM, and therefore call into question whether the low level of *Nodal* we detect at the node in some of our *Zic2* mutants is the only reason for their failure to induce NODAL downstream targets in the LPM. Assessing the exact contribution of the loss of *Nodal* expression at the node to the observed phenotype would require a rescue experiment in which a transgenic construct is used to drive *Nodal* expression at the node in a *Zic2*^*iso*^ mutant background.

The situation is arguably complex in these mutants, because *Nodal* may not be the only gene in the pathway regulated by ZIC2. The level of *Nodal* expression in the node required to induce its own expression in the LPM may thus be different, and perhaps higher, than that in wildtype embryos, to compensate for a possible concomitant down-regulation of partners or agonists. *Gdf1*, encoding a co-ligand of NODAL, is likewise expressed in perinodal cells where it is required to ensure the adequate transfer of NODAL to the LPM^60^, and could be one such partner as its absence from the node also leads to right isomerism. No ZIC2-binding peaks are present at the *Gdf1* locus^26,27^ and our microarray analyses of *Zic2*^*iso*^ mutant embryos detected no alteration of *Gdf1* expression (Table S2), but, given that *Gdf1* is also bilaterally expressed in lateral plate mesoderm at this stage, this experiment may not be sensitive enough to detect its specific misregulation in perinodal cells. Interestingly, the expression of *Gdf1* in these cells, like that of *Nodal* and *Cerl2*, is known to be dependent on NOTCH signalling^61^. Our observation that in most *Zic2*^*iso*^ mutant embryos *Cerl2* shows normal levels of expression in the node indicates that NOTCH signalling is likely to be intact in these mutants. This, together with a previous report that absence of *Nodal* expression in the node does not affect the expression of *Gdf1* there^58^ make it quite possible that its expression is similarly unaffected in the node of *Zic2*^*iso*^ mutant embryos. However, further investigations are necessary to make certain that this is case, not just for *Gdf1* but also for all the genes expressed at the node that contribute to the production and propagation of the left identity-inducing signal.

The occurrence of right isomerism has been described in embryos carrying point mutations in either *Pkd1l1* or *Pkd2*, which are believed to affect the detection of nodal flow by immotile cilia^62^, rather than affecting motile cilia function (node morphology and cilia motility is unaffected in these mutants). Crucially, not only does the NODAL signalling cascade fail to be activated in the LPM of these mutants, both *Nodal* and *Cerl2* expression at the node remain symmetrical, unlike in *Zic2* mutants. *Pkd1l1* and *Pkd2* expression is restricted to the node at E7.0–E7.75. Our microarray data shows no evidence for a downregulation of their transcripts in the *Zic2*^*iso*^ mutants, but *in situ* data suggests they may be reduced in *Zic2*^*Ku*^^24^ mutants. The anomalies we detected in the laterality of *Nodal* and *Cerl2* expression around the node of *Zic2*^*iso*^ mutant embryos confirmed that their nodal flow is perturbed, and suggest that motile cilia at the node present defects similar to those characterized in *Zic2*^*Ku*^ mutants^24^. However, the emergence of these anomalies, and their occasional consistency with corresponding anomalies in the expression of *Nodal* or *Pitx2* in the LPM, suggest immotile cilia are functional and argue against this mutant version of ZIC2 having a major impact downstream of the nodal flow.

The expression of *Cerl2* in perinodal cells of *Zic2*^*iso*^ mutant embryos indicates NOTCH signalling is intact. This suggests that the *Nodal* locus in this mutant is either unresponsive to NOTCH signalling or unable to maintain its own expression after it is induced. The dynamic expression of *Nodal* is regulated by five distinct enhancers, three of which (NDE, PEE and HBE) were found to harbour significant ZIC2-binding peaks in ESCs and/or in EpiSCs (Fig. 5a). NDE is active in perinodal cells and its deletion eliminates most, but not all, of *Nodal* expression at the node. Its transcriptional activity has been shown to be dependent on NOTCH signalling^63^ and a previous study using *Xenopus* cells, has shown that it can be activated by ZIC3^54^, a transcription factor closely related to ZIC2. NDE therefore would appears as an ideal candidate to mediate the influence of ZIC2 on *Nodal* expression at the node, however we did not detect an effect of ZIC2 on its transcriptional activity in our luciferase assay and further analysis will be necessary to find out whether it does mediate this influence *in vivo*.

PEE transcriptional activity is detected in the proximal epiblast and in the anterior primitive streak, but not in the node^50^. Deletion of PEE has been shown to result in a range of defects, including heart abnormalities^50^, which are reminiscent of those of *Zic2* mutants. However, like for NDE, we did not detect an effect of ZIC2 on the transcriptional activity of PEE in our luciferase assay. These data leave open the possibility that this enhancer mediates the influence of ZIC2 on a domain of *Nodal* expression that is critical for anterior patterning and the establishment of laterality, but again further analysis will be necessary to confirm this is the case.

HBE was the only *Nodal* enhancer showing a ZIC2-mediated transcriptional activation response. Mutagenesis analysis indicated both that a defined ZIC2-binding site within HBE, ZBS3, is required for this response, and that a protein carrying the same mutation as the *iso* mouse shows a reduced ability to activate HBE. While the transcriptional activity of HBE is highest at preimplantation stages it is still detectable in the post-implantation epiblast, in the primitive streak and in the early node^49^. The impact on embryonic development of its deletion at these stages is not yet known, but these data suggest that an interaction between ZIC2 and HBE may be required for *Nodal* to be correctly expressed at the node. Testing this hypothesis calls for an investigation of the impact of a targeted mutation of ZBS3 on *Nodal* expression and the establishment of laterality.

Our analyses indicate that ZBS3 is a low-affinity ZIC2-binding site, and that higher affinity ZIC2-binding sites in HBE are dispensable for its transcriptional activity. This is consistent with current models of how transcription factors regulate gene expression, which place greater emphasis on the critical role played by low-affinity binding sites^64^. This result led us to consider the possibility that another *Nodal* enhancer, ASE, in which a low-affinity ZIC2-binding site similar to ZBS3 was identified in EpiSCs, might also mediate the influence of ZIC2 on *Nodal* expression at the node. ASE is an auto-regulatory enhancer, known to be dependent on Activin/Nodal signalling. Mouse embryos deleted for ASE show very weak *Nodal* expression in the left LPM, which leads to partial right isomerism later on^47^. Crucially, the level of *Nodal* expression at the node at E7.5 and E8.5 in these mutants is similar to that of wildtype embryos, except that it remains symmetrical. This observation appears to rule out the possibility that ASE plays a critical role in mediating the influence of ZIC2 on *Nodal* node expression.

Thus, a review of the evidence relating to NDE, PEE and HBE leaves open the possibility that they may all be, directly or indirectly, involved in promoting the influence of ZIC2 on the expression of *Nodal* during the time window in which ZIC2 function at the midline is critical for the establishment of a left identity in the LPM, which broadly extends from E6.5 to E7.5. The expression of *Zic2* and the transcriptional activity of NDE only overlap briefly in the young node, which seems to preclude a prolonged interaction. In contrast, the overlap with the transcriptional activities of HBE and PEE in the epiblast and in the primitive streak, ahead of node formation, is extensive. This may be important as ZIC2 has been shown to act as a pioneer factor in ESCs, binding to target genes before they are transcriptionally active, seeding the locus to facilitate binding of additional factors later in more differentiated cell types^26,29^. Furthermore, in the case of *Nodal*, the presence of one of the ZIC2-bound enhancers, HBE, has been shown to condition the later activation of at least one of the other *Nodal* enhancers^49^. These observations may explain how the impact of ZIC2 absence on *Nodal* node expression could be delayed until a time when the transcription factor is no longer expressed. ZIC2 associates at a subset of loci in ESCs with the NURD chromatin remodelling complex^27^, a complex known to regulate bivalent enhancers. ZIC2 is bound to the *Nodal* locus in epiblast stem cells, precursors of the node, and is able to activate expression in *in vitro* assays from one such bound enhancer (HBE), yet there is no obvious phenotype at this stage of development in *Zic2* mutants. All available data is therefore compatible with the possibility that ZIC2 interaction with regulatory sequences at the *Nodal* locus in node cell precursors in the primitive streak primes it for later activation, a pre-requisite for the correct establishment of left-sided identity. Further work will be required to test this hypothesis and to elucidate the precise molecular mechanisms by which ZIC2 may regulate *Nodal* expression.

Our analysis lends support to the hypothesis that cardiovascular and other visceral defects seen in ZIC2 HPE patients may result from an underlying laterality defect. However, it would appear that the co-morbidity of cardiovascular laterality defects with HPE is a relatively rare event in reported *ZIC2* HPE cases, occurring in only between 9 and 14% of cases^2,3^. Cardiovascular malformations of this kind are embryonic lethal in mice and thus many pregnancies may be lost before term, indeed only one of the probands we describe was born alive. Thus, many cases may go unreported. It should also be noted that most patients have heterozygous *ZIC2* mutation; heterozygosity in mice is associated with mild neural tube defects but is not associated with cardiovascular malformations. Thus, milder forms of HPE reported in living patients would not be expected to be associated with cardiovascular anomalies. *Situs* abnormalities do not always manifest in right isomerism in human cases as in the mouse model, and we find evidence for discordance between cardiac and pulmonary *situs* suggesting heterotaxy in one patient. This is likely to be for a number of reasons. The patient with heterotaxy (Proband 7) carries a large chromosomal deletion encompassing many genes in addition to *ZIC2* and therefore genetic interactions are likely to be a factor. Genetic background is known to affect the penetrance of cardiovascular laterality phenotypes^32^, indicating that genetic interactions at multiple loci influence the phenotype. This may also reflect the influence of environmental factors, which are known to impact upon the expression of cardiovascular phenotypes^65^. Finally, in many cases it is not possible to make a definitive diagnosis based on the information provided.

Our data nevertheless explain the co-morbidity of holoprosencephaly with congenital heart disease and suggest that *ZIC2* should be considered as a candidate for screening for the latter disease.

## Methods

### Human genetics

All protocols were approved by the local ethics committee of Rennes Hospital. All work was carried out in accordance with this protocol. All samples were obtained and analysed with informed consent according to the protocols approved by the local ethics committee (Rennes Hospital).

### Mouse genetics

All animal procedures were approved by the Committee for Animal Care and Ethical Review at the University of Oxford, and all the experiments conformed to the UK Animals (Scientific Procedures) Act, 1986, incorporating Directive 2010/63/EU of the European Parliament. All animal procedures were performed in accordance with UK Home Office regulations (PPL 30/3174). The *iso* allele was isolated in a random recessive ENU mutagenesis screen in which cardiovascular anomalies were identified by MRI screening of E14.5 embryos^35,36^. Male C57Bl6/J males were mutagenized then crossed to C3H/HeH females. G3 progeny were screened for a phenotype. TALEN alleles of *Zic2* have been previously described^31^. All *Zic2* alleles were subsequently backcrossed for several generations and then maintained on a C3H/HeH background (mice obtained from MRC Harwell, Oxfordshire, UK) and embryos of both sexes were used in experiments. Heterozygous animals were crossed and pregnant dams were sacrificed by cervical dislocation before the embryos were dissected and processed for further analyses. Genotyping was performed using allele-specific Taqman probes on DNA obtained from ear biopsies. In all experiments, mutant embryos were compared to littermate controls.

### Genetic mapping and sequencing

Mutations were mapped using a panel of SNP markers that differentiate between C57BL6/J and C3H/HeH mouse strains, as described previously^35^, identifying a minimal homozygous segregating interval lying on chromosome 14 between SNP rs13482392 (118.9 Mb) and the end of the chromosome at 124 Mb. The identified chromosomal interval was then exome sequenced using SureSelectXT mouse all exon kit (Agilent, as recommended by the manufacturer). Captured libraries were sequenced on the Illumina platform as paired-end 76-bp reads. Study accession number ERP000530. The only exonic mutation identified within this interval was a C to A substitution at position 122877892 indicating a stop-gain mutation (Y401X) in the *Zic2* gene disrupting the fifth zinc finger domain (Fig. S1; Table S1). This was confirmed by capillary sequencing. No other exonic mutations were identified within the mapped interval.

### Phenotyping by MRI/µCT scanning

Embryos were harvested at either E14.5 or E15.5, exsanguinated in warm HANKS saline (SIGMA, H4641), cooled down in ice cold PBS and then fixed in 4% paraformaldehyde. Imaging was performed using either MRI (*Iso, A8, A10/17, A19*) or by µCT (*A5*). MRI was performed as previously reported^66^. µCT was performed using SkyScan 1172 scanner (Kontich, Belgium). Prior to scanning, embryos were incubated in 0.025 N Lugol’s solution for 4 days to achieve soft tissue contrast and embedded in a tube with 1% agarose. Generated datasets were analysed and 3D reconstructions generated using Amira 5.3.3 software (FEI Visualization Sciences Group, Merignac, France).

### Microarray analysis

E8.5 wildtype and *Zic2*^*iso/iso*^ whole embryos were collected and snap frozen in liquid nitrogen. Eight stage-matched embryos were pooled per sample and three replicates of each genotype were performed. RNA was prepared using the RNeasy Plus Micro kit (Qiagen) and hybridised to Illumina Mouse WG6 v2 arrays at Oxford Genomics Centre (following manufacturers’ protocol). Raw data were imported into R statistical software for processing and analysis (http://www.R-project.org). Pre-processing and normalisation steps were performed with the BioConductor^67^ package ‘Variance Stabilisation and Normalisation’ (VSN)^68^. Quality control analyses showed the data were high quality with no outlier samples. Statistical analysis was performed on the full dataset (approximately 45,000 probes) with the Linear Models for Microarray Analysis (LIMMA) package^69^. Raw p-values were corrected for multiple testing using the false discovery rate (FDR) controlling procedure^70^. At 5% FDR, this resulted in 30 significantly changed genes. A heatmap was generated using the Expander programme^71^. Microarray data has been submitted to the Gene Expression Omnibus (accession number will be provided following acceptance).

### qPCR

Total RNA was harvested from whole embryos as above. RNA was reverse transcribed using the Quantitect reverse transcription kit with genomic DNA eliminator (Qiagen), and analysed by SYBR Green based qPCR (Fig. 2c; Supplementary Table 3 lists primers used) or using Taqman probes (Figs 2d, S4). Graphs show the mean +/− standard deviation for three biological replicates, each consisting of a pool of embryos. A one tailed t-test was performed on linear expression values normalised to GAPDH to test the hypothesis that expression of selected genes is reduced in the mutant relative to wildtype.

### In situ hybridisation

Embryos were harvested at E8.5 and fixed in 4% paraformaldehyde, 0.2% glutaraldehyde for 3 hours at 4 °C before being dehydrated and stored in methanol at −20 °C until use. Prior to hybridising, embryos were bleached in 6% H_2_O_2_ and digested for 6 minutes in 10 µg/ml proteinase K. Hybridisation was performed with digoxigenin-labelled RNA probes (2 µg/ml) at 70 °C following standard protocols^72^. Probes used are listed in Supplementary Table 4.

### Chip-Seq analysis

ChIP-seq data used in this paper are from published studies^26,27^; GEO accession numbers GSE61188, GSE74636. Binding peaks were visualised using the Integrated Genome Viewer software^73^.

### Luciferase assays

All assays were performed with U2-OS cells (obtained from ATCC) grown in DMEM with 10% FBS. Cells were tested and confirmed free of mycoplasma contamination. Luciferase reporters carrying *Nodal* enhancers linked to a viral E1b promoter have been previously described^49^. Generation of Y401X ZIC2 and deletion of ZIC2 binding sites within HBE was performed by site directed mutagenesis (NEB) using primers listed in Supplemental Table 5. Cells were transfected using Fugene reagent (Promega) with either HA-ZIC2 (Zic2), HA-ZIC3 (Zic3), Y401X ZIC2 (Zic2-iso) or an empty vector (pcDNA) along with a 5× excess of reporter plasmid. A Renilla luciferase reporter was co-transfected to control for transfection efficiency. Cells were harvested after 48–72 hrs and assayed using the Dual Luciferase Reporter assay system (Promega). Each data point represents the mean of three technical replicates performed during the same experiment, and each experiment was repeated three times, as shown on the graphs. Asterisks indicate results of a one tailed t-test of samples with unequal variance testing the null hypothesis either that transfection with ZIC2 does not result in a change in activation of a given reporter over transfection with pcDNA (Fig. 5b,d) or that there is no difference between the two indicated conditions (Fig. 5c). *p < 0.05, NS = not significant.

### EMSA assays

Crude protein extracts were derived from HEK293T cells (obtained from ATCC, tested for mycoplasma contamination) transfected with a Zic2-HA expression plasmid or a control pcDNA plasmid. Cells were lysed in a solution of 10 mM HEPES pH7.9, 1.5 mM MgCl2, 10 mM KCl, 0.5 mM DTT and nuclei pelleted by centrifugation. Nuclei were lysed in 20 mM HEPES pH7.9, 25% glycerol, 0.42 M NaCl, 1.5 mM MgCl2, 0.2 mM EDTA, 0.5 mM DTT. Short 31 bp double-stranded biotin labelled probes (listed in Supplemental Table 6) were synthesised and EMSA assays performed using the Lightshift Chemiluminescent EMSA kit (Life Technologies). Unlabelled oligos were used as competitors and a supershift was performed with a monoclonal αHA antibody (Covance #MMS-101R).

### Data availability

The microarray datasets generated in the current study are available in the GEO repository, accession number GSE106350. Luciferase constructs and mouse alleles described in this work are available on request.

## Electronic supplementary material

Supplementary data
