# Supplementary material for: A Requirement for Zic2 in the Regulation of Nodal Expression Underlies the Establishment of Left-Sided Identity
Source: Sci Rep. 2018 Jul 11;8:10439. doi: 10.1038/s41598-018-28714-1 (PMC6041270; doi:10.1038/s41598-018-28714-1)
Supplement: Supplementary file 1 — Supplementary data [file 41598_2018_28714_MOESM1_ESM.docx]

**A REQUIREMENT FOR ZIC2 IN THE REGULATION OF NODAL EXPRESSION UNDERLIES THE ESTABLISHMENT OF LEFT-SIDED IDENTITY**

Iain M. Dykes, Dorota Szumska, Linta Kuncheria, Rathi Puliyadi, Chiann-mun Chen, Costis Papanayotou, Helen Lockstone, Christèle Dubourg, Véronique David, Jurgen E. Schneider, Thomas M. Keane, David J. Adams, Steve D. M. Brown, Sandra Mercier, Sylvie Odent, Jérôme Collignon, Shoumo Bhattacharya

**Supplementary Data**


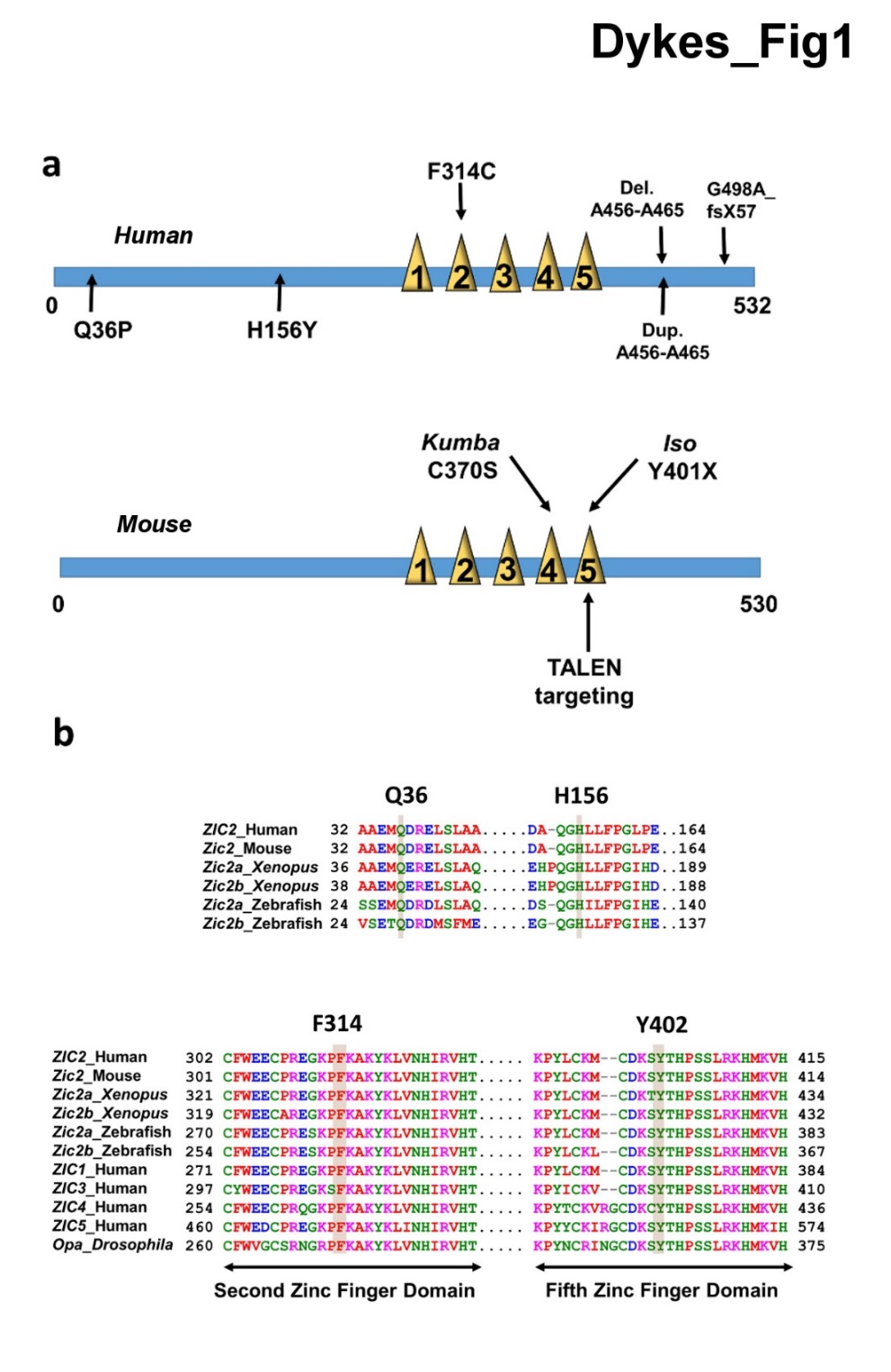


Supplementary Figure 1. Mutations of *ZIC2* observed in probands with holoprosencephaly and cardiovascular or laterality defects

a. Cartoons to illustrate human ZIC2 protein (upper) and mouse Zic2 protein (lower). The five zinc finger domains are shown in yellow. The location of amino acids affected in probands with a putative laterality defect are shown in the upper image, while mouse mutant alleles are shown in the lower.

b. CLUSTAL-W2 protein alignment to show conservation of amino acids mutated in human (Q36, H156 and F314) or in mouse (Y402; equivalent to Y401 in mouse). Accession numbers are in the supplement.

WT CCTATCTCTGCAAGATGTGTGACAAGTCCTACACGCATCCCAGCTCGTTGCGGAAGCACA

Iso CCTATCTCTGCAAGATGTGTGACAAGTCCTAAACGCATCCCAGCTCGTTGCGGAAGCACA

A5 CCTATCTCTGCAAGATGTGTGACAAGTCC-----------CAGCTCGTTGCGGAAGCACA

A8 CCTATCTCTGCAAGATGTGTGACAAGTCCTA-ACGCATCCCAGCTCGTTGCGGAAGCACA

A10/17 CCTATCTCTGCAAGATGTGTGACAA--CCTACACGCATCCCAGCTCGTTGCGGAAGCACA

A19 CCTATCTCTGCAAGATGTGTGACAAGTCC------------CGCTCGTTGCGGAAGCACA

**Zinc Finger 4 Zinc Finger 5**

WT THTGEKPFQCEFEGCDRRFANSSDRKKHMHVHTSDKPYLCKMCD---KSYTHPSSLRKHMKVHESS 418

ISO THTGEKPFQCEFEGCDRRFANSSDRKKHMHVHTSDKPYLCKMCD---KS*---------------- 401

A5 THTGEKPFQCEFEGCDRRFANSSDRKKHMHVHTSDKPYLCKMCD---KSQLVAEAHEGP*------ 411

A8 THTGEKPFQCEFEGCDRRFANSSDRKKHMHVHTSDKPYLCKMCD---KS*---------------- 401

A10/17 THTGEKPFQCEFEGCDRRFANSSDRKKHMHVHTSDKPYLCKMCDNLHASQLVAEAHEGP*------ 414

A19 THTGEKPFQCEFEGCDRRFANSSDRKKHMHVHTSDKPYLCKMCD---KS----RSLRKHMKVHESS 414

Supplementary Figure 2. *Zic2* targeted mutant alleles used in this study.

DNA (upper) and amino acid sequence (lower) of wildtype mouse *Zic2* (top row), the *iso* mutant (second row) and the five mutants generated by TALEN-mediated genome editing ^21^. All mutations disrupt the fifth DNA-binding zinc finger domain by introducing a premature stop codon (*) and/or a frame shift error (red) or by deleting sequence.


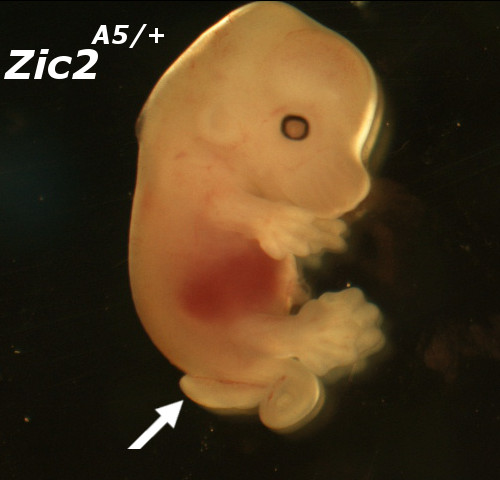


Supplementary Figure 3. *Zic2* heterozygous phenotype

*Spina bifida* was seen in 2 of 27 heterozygote embryos (arrow). This embryo also has a curly tail phenotype.


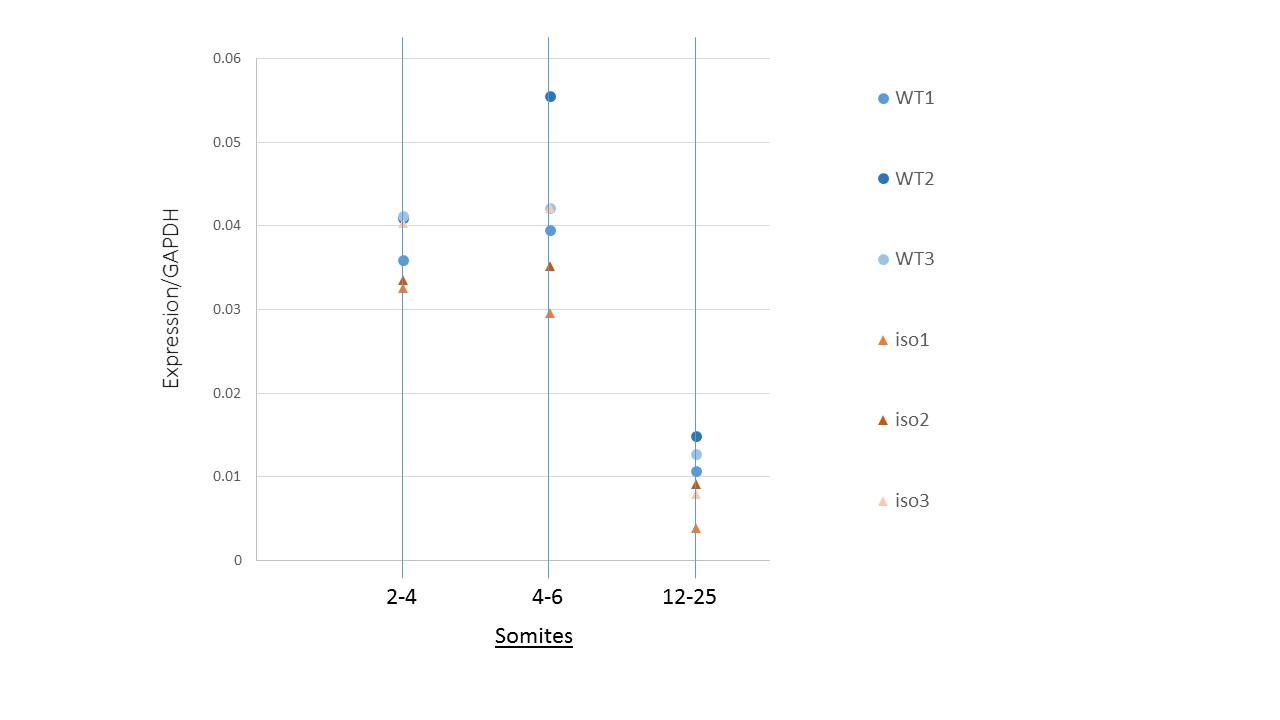


Supplementary Figure 4. *Zic2* mRNA is expressed in the *iso* mutant embryo

Taqman qRT-PCR analysis of *Zic2* expression was performed at three different ages (2-4 somites, 4-6 somites and 12-25 somites) in wildtype (WT) and *Zic2^iso^* mutant embryos. Three independent biological replicates of pooled embryos were performed for each condition.

**
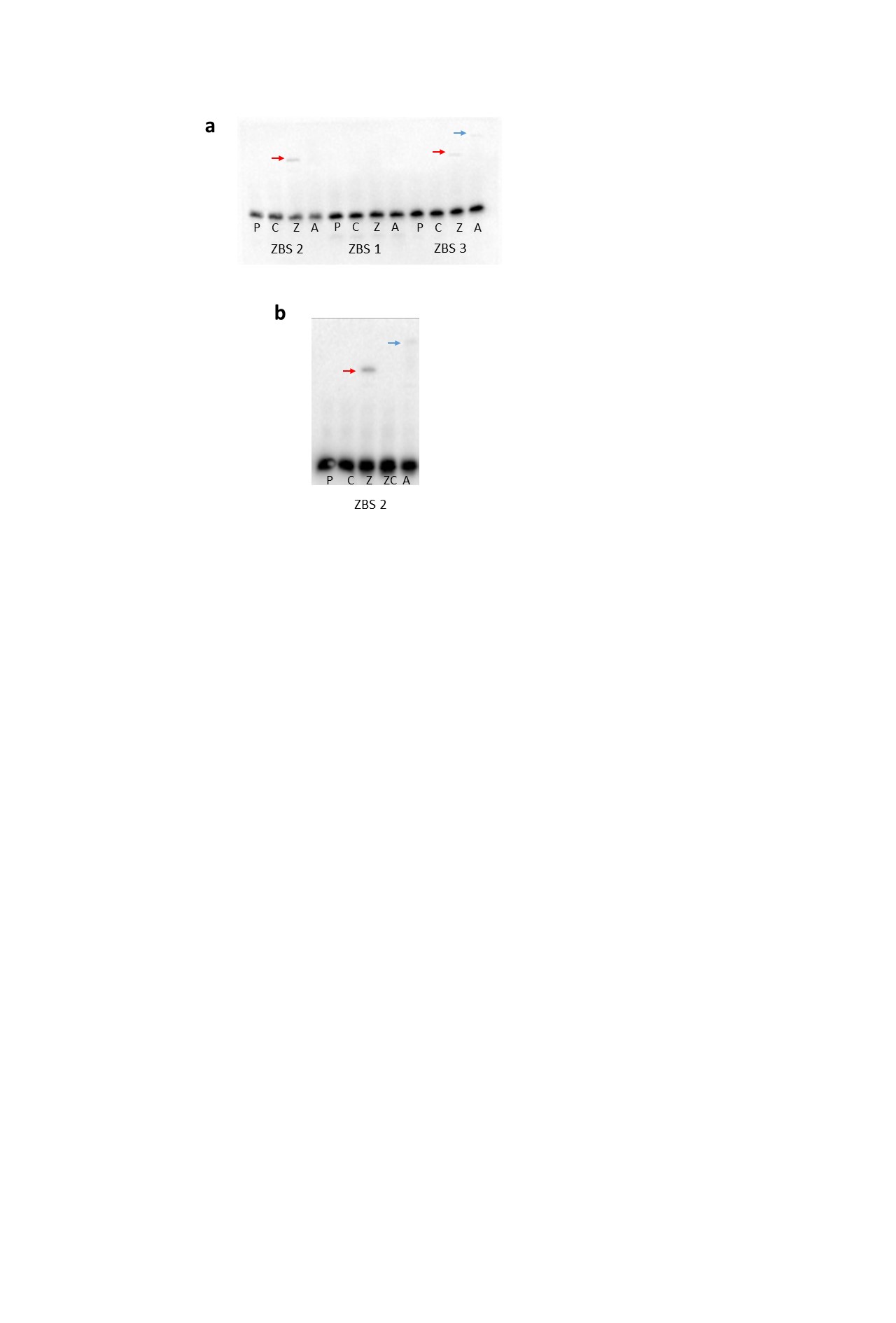
**

**Supplementary Figure 5. ZIC2 binds to sites within the HBE enhancer**

Complete gel pictures of EMSA assays shown in Fig 5.

1. Blot showing comparison of sites ZBS1,2 and 3 run on the same gel.
2. Blot showing site ZBS2

Abbreviations: P =probe only, Z = probe + HA-ZIC2, ZC = probe + HA-ZIC2 + unlabelled competitor, ZA = probe + HA-ZIC2+ αHA antibody. Red arrow = gel shift, blue arrow = antibody supershift.

**Supplementary Table 1**. Mutations identified by exome capture in the *iso* mutant on chromosome 14 within the interval 118.9 Mb to 124Mb. A Stop_Gain within the gene *Zic2* is the only exonic mutation identified.

| **Chromosome** | **Position** | **Depth** | **Consequence** | **Gene name** |
| --- | --- | --- | --- | --- |
| 14 | 119015695 | 90 | INTRONIC+ENSMUST00000166646 | ABCC4 |
| 14 | 121693660 | 17 | INTRONIC | STK24 |
| 14 | 121693663 | 17 | INTRONIC | STK24 |
| 14 | 122061644 | 73 | INTRONIC+ENSMUST00000100298 | DOCK9 |
| 14 | 122777157 | 50 | INTRONIC+ENSMUST00000110684 | CLYBL |
| 14 | 122876030 | 79 | INTRONIC+ENSMUST00000110682 | ZIC2 |
| 14 | 122877892 | 198 | STOP_GAINED | ZIC2 |
| 14 | 123697165 | 68 | INTRONIC | NALCN |
| 14 | 123698546 | 44 | INTRONIC | NALCN |
| 14 | 123747918 | 48 | INTRONIC+ENSMUST00000072254 | NALCN |

**Supplementary Table 2**. Gene expression changes in the 0-4 somite *Zic2 iso* mutant assayed by microarray analysis.

| **Probe_Id** | **Gene_Name** | **Symbol** | **Mean Expression** | | | | **Fold Change** | **Adjusted p-value** | |
| --- | --- | --- | --- | --- | --- | --- | --- | --- | --- |
|  |  |  | **KO** | | **WT** | |  |  |  |
| **Downregulated:** | | | | | | | | | |
| ILMN_2744317 | forkhead box D4 | Foxd4 | 273 | | 1819 | | -6.68 | 0.0000 | |
| ILMN_2713164 | left-right determination factor 2 | Lefty2 | 84 | | 431 | | -5.12 | 0.0000 | |
| ILMN_2776165 | nodal | Nodal | 163 | | 408 | | -2.50 | 0.0002 | |
| ILMN_2733155 | Fez family zinc finger 1 | Fezf1 | 470 | | 990 | | -2.11 | 0.0002 | |
| ILMN_1249941 | growth differentiation factor 10 | Gdf10 | 207 | | 416 | | -2.01 | 0.0004 | |
| ILMN_2635708 | family with sequence similarity 183 B | Fam183b | 222 | | 442 | | -1.99 | 0.0001 | |
| ILMN_2610013 | sonic hedgehog | Shh | 335 | | 652 | | -1.95 | 0.0006 | |
| ILMN_1232648 | goosecoid homeobox | Gsc | 180 | | 348 | | -1.94 | 0.0002 | |
| ILMN_2488690 | LOC102632564 ncRNA | XR_377376 | 132 | | 252 | | -1.91 | 0.0004 | |
| ILMN_1215019 | dynein light chain roadblock-type 2 | Dynlrb2 | 122 | | 232 | | -1.89 | 0.0002 | |
| ILMN_1246073 | left right determination factor 1 | Lefty1 | 120 | | 220 | | -1.83 | 0.0457 | |
| ILMN_3161263 | frizzled homolog 5 (Drosophila) | Fzd5 | 872 | | 1566 | | -1.80 | 0.0008 | |
| ILMN_2776047 | engrailed 1 | En1 | 718 | | 1265 | | -1.76 | 0.0037 | |
| ILMN_1227587 | myocardial infarction associated transcript (ncRNA) | Miat | 227 | | 384 | | -1.69 | 0.0031 | |
| **Probe_Id** | **Gene_Name** | **Symbol** | **Mean Expression** | | | | **Fold Change** | **Adjusted p-value** | |
|  |  |  | **KO** | | **WT** | |  |  |  |
|  |  |  |  | |  | |  |  | |
| ILMN_2878542 | myosin, light polypeptide 1 | Myl1 | 568 | | 960 | | -1.69 | 0.0457 | |
| ILMN_2778151 | SRY-box containing gene 9 | Sox9 | 1543 | | 2546 | | -1.65 | 0.0051 | |
| ILMN_2495167 | fibronectin leucine rich transmembrane protein 1 | Flrt1 | 160 | | 255 | | -1.59 | 0.0217 | |
| ILMN_2765047 | chordin | Chrd | 246 | | 377 | | -1.53 | 0.0214 | |
| ILMN_2744927 | calcitonin/calcitonin-related polypeptide, alpha | Calca | 207 | | 316 | | -1.53 | 0.0279 | |
| ILMN_2515816 | claudin 10 | Cldn10 | 300 | | 457 | | -1.53 | 0.0157 | |
| ILMN_2620406 | neuronal pentraxin 2 | Nptx2 | 309 | | 467 | | -1.51 | 0.0390 | |
| ILMN_1249022 | calneuron 1 | Caln1 | 197 | | 298 | | -1.51 | 0.0457 | |
| ILMN_3007428 | SRY-box containing gene 9 | Sox9 | 134 | | 202 | | -1.51 | 0.0279 | |
| **Upregulated:** |  |  |  | |  | |  |  | |
| ILMN_2925169 | Unknown | N/A | 432 | | 196 | | 2.21 | 0.0207 | |
| ILMN_2649333 | ribosomal protein S6 kinase-like 1 | Rps6kl1 | 376 | | 207 | | 1.82 | 0.0028 | |
| ILMN_2870549 | DnaJ (Hsp40) homolog, subfamily C, member 3 | Dnajc3 | 863 | | 499 | | 1.73 | 0.0022 | |
| ILMN_2706623 | Nanog, transcript variant X1, X2 | Nanog | 266 | | 159 | | 1.67 | 0.0167 | |
| ILMN_3158250 | chemokine (C-X-C motif) ligand 12 | Cxcl12 | 470 | | 293 | | 1.60 | 0.0485 | |
| ILMN_2955047 | desmoglein 2 | Dsg2 | 276 | | 176 | | 1.57 | 0.0279 | |
| **Probe_Id** | **Gene_Name** | **Symbol** | **Mean Expression** | | | **Fold Change** | | | **Adjusted p-value** |
|  |  |  | **KO** | **WT** | |  |  |  |  |
| ILMN_1230366 | doublesex and mab-3 related transcription factor 3 | Dmrt3 | 215 | | 146 | | 1.48 | 0.0457 | |
|  |  |  |  | |  | |  |  | |
| **Selected genes of interest:** | |  |  | |  | |  |  | |
| ILMN_3118071 | paired-like homeodomain transcription factor 2 | Pitx2 | 1473 | | 2191 | | -1.49 | 0.0712 | |
| ILMN_2754305 | DAN domain family, member 5 | Dand5 | 41 | | 42 | | -1.03 | 0.9710 | |
| ILMN_7610754 | zinc finger protein of the cerebellum 2 | Zic2 | 153 | | 213 | | -1.39 | 0.5070 | |
| ILMN_5260546 | zinc finger protein of the cerebellum 3 | Zic3 | 1596 | | 1554 | | 1.03 | 0.9882 | |
| ILMN_2600546 | polycystic kidney disease 1 like 1 | Pkd1l1 | 80 | | 71 | | 1.12 | 0.9201 | |
| ILMN_2866327 | polycystic kidney disease 2 | Pkd2 | 699 | | 820 | | -1.17 | 0.8395 | |
| ILMN_1237300 | notochord homolog (Xenopus laevis) | Noto | 102 | | 80 | | 1.29 | 0.7008 | |
| ILMN_1225333 | notochord homolog (Xenopus laevis) | Noto | 98 | | 89 | | 1.10 | 0.9462 | |
| ILMN_2833082 | notochord homolog (Xenopus laevis) | Noto | 117 | | 118 | | -1.01 | 0.9961 | |
| ILMN_1236725 | growth differentiation factor 1 | Gdf1 | 2849 | | 2907 | | -1.02 | 0.9907 | |
| ILMN_1247691 | hairy and enhancer of split 1 (Drosophila) | Hes1 | 1096 | | 1161 | | -1.06 | 0.9709 | |
| ILMN_1253414 | hairy and enhancer of split 5 (Drosophila) | Hes5 | 184 | | 166 | | 1.11 | 0.5500 | |
| ILMN_1241915 | Notch gene homolog 1 (Drosophila) | Notch1 | 277 | | 266 | | 1.04 | 0.9881 | |
| ILMN_2678002 | Notch gene homolog 2(Drosophila) | Notch2 | 87 | | 84 | | 1.04 | 0.9805 | |
| ILMN_2697380 | Notch gene homolog 3 (Drosophila) | Notch3 | 438 | | 403 | | 1.09 | 0.9523 | |
| ILMN_1220697 | Notch gene homolog 4(Drosophila) | Notch4 | 457 | | 529 | | -1.16 | 0.8873 | |
| ILMN_2721188 | delta-like 1 (Drosophila) | Dll1 | 286 | | 316 | | -1.10 | 0.9404 | |
| ILMN_2643777 | delta-like 3 (Drosophila) | Dll3 | 105 | | 94 | | 1.12 | 0.9353 | |
| ILMN_2588315 | recombination signal binding protein for immunoglobulin kappa J region | Rbpj | 113 | | 100 | | 1.13 | 0.9076 | |

**Supplementary Table 3**. SYBR Green qPCR primers used in this study.

| **Primer** | **Sequence** | **Primer** | **Sequence** |
| --- | --- | --- | --- |
| ChrdF | ATTACCTGCAGATGTGGGGC | SHHF | CACCCCCAATTACAACCCCG |
| ChrdR | CTCAGAGGACCTTTGGGCTG | SHHR | CTTGTCTTTGCACCTCTGAGTC |
| Dynlrb2F | GCGCGATGACAGAAGTGGAG | Sox9F | GCCACGGAACAGACTCACAT |
| Dynlrb2R | TCCAGGGTTGTTCGGATTGG | Sox9R | GGACCCTGAGATTGCCCAGA |
| En1F | CCGGTGGTCAAGACTGACTC | NodalF | CTGGCGTACATGTTGAGCCT |
| En1R | CTGGTGCGTGGACCAGAG | NodalR | GGTCACGTCCACATCTTGCG |
| Fam183bF | CTCAAAGAGCTACGGGCACA |  |  |
| Fam183bR | AGGTTCAGGAACTTGGCCTT |  |  |
| FezF1F | CGCCCAACTTTTGTCGGAAA |  |  |
| FezF1R | CGCATTAAAGACCTTGCCGC |  |  |
| Foxd4F | CGCCAAAGTCAGTACTGGGA |  |  |
| Foxd4R | GCGGGCTCTGTTGATGTTTC |  |  |
| FZD5F | GAAGAGAAGGCGAGTGACCG |  |  |
| FZD5R | AAGGACAGAACTCTGTGGCG |  |  |
| GapdhF | GTGAAGGTCGGTGTGAACGG |  |  |
| GapdhR | AGTTGAGGTCAATGAAGGGG |  |  |
| GDF10F | GCAGACATCGGGTGGAATGA |  |  |
| GDF10R | GGACAATCTTGGGCATGGGG |  |  |
| GscF | GGAGACGAAGTACCCAGACG |  |  |
| GSCR | AAACCAGACCTCCACCTTCTC |  |  |
| Lefty1F | CCCAAGGATGTGCCTTTCAT |  |  |
| Lefty1R | CAGGGATTGCCCATTAGGGAG |  |  |
| Lefty2F | TGTAGTCTCCCTGTCCACAGAT |  |  |
| Lefty2R | AGCAAAGGTCTGACGAGAGC |  |  |
| MIATF | TCCGGCAGAAATGAATGGCT |  |  |
| MIATR | CCCCATAGCAACCAAGCTCA |  |  |

**Supplementary Table 4**. *In situ* probes used in this paper.

| **Probe** | **Corresponding Refseq Nucleotide coordinates** | **PCR/Plasmid** | **Source** | **Reference** | **Notes** |
| --- | --- | --- | --- | --- | --- |
| *Nodal* | 550-1099 | PCR | own | n/a | Similar to probe used by Zhou et al., 1993 |
| *Lefty2* | 1163-1695 | Plasmid | Hamada lab | Meno et al., 1997 |  |
| *Dand5* | 456-1561 | Plasmid | Belo lab | Marques et al., 2004 |  |
| *Pitx2* | 14-1759 of Pitx2a | Plasmid | Izpisua-Belmonte lab | Ryan et al., 1998 | This probe cross reacts with both Pitx2b and Pitx2c. From 294-1759 the sequence is identical to Pitx2c. |
| *Shh* | 200-850 | Plasmid | McMahon lab | Echelard et al., 1993 |  |

Supplementary Table 5. Site directed mutagenesis primers

| Oligo | Sequence |
| --- | --- |
| ISO-F | ACAAGTCCTAAACGCATCCCAG |
| ISO-R | CACACATCTTGCAGAGATAGG |
| ZBS1-F | AGAGGCTGGCAACCCCAG |
| ZBS1-R | AGGTCCAGGTGGCTAAGG |
| ZBS2-F | CTCGATGAATATGTAGATAGC |
| ZBS2-R | TTCTTATCTGAAGGCTTTGG |
| ZBS3-F | ACTTTGGTATTTGAAGCTG |
| ZBS3-R | GAGCAAGGGGAATAATTAC |

Supplementary Table 6. EMSA oligo probes

| Oligo | Sequence |
| --- | --- |
| ZBS1-F | ACCTGGACCTCCCAGGAGGTGAGAGGCTGGC |
| ZBS1-R | GCCAGCCTCTCACCTCCTGGGAGGTCCAGGT |
| ZBS2-F | CAGATAAGAA CCCCTGGGGTG CTCGATGAAT |
| ZBS2-R | ATTCATCGAGCACCCCAGGGGTTCTTATCTG |
| ZBS3-F | CCCCTTGCTCCCCAGGAGGGCACTTTGGTAT |
| ZBS3-R | ATACCAAAGTGCCCTCCTGGGGAGCAAGGGG |
